# Sources and Delivery of Nutrients to the Northwestern Gulf of Mexico from Streams in the South-Central United States^1^

**DOI:** 10.1111/j.1752-1688.2011.00583.x

**Published:** 2011-10

**Authors:** Richard A Rebich, Natalie A Houston, Scott V Mize, Daniel K Pearson, Patricia B Ging, C Evan Hornig

**Keywords:** nutrients, nonpoint source pollution, transport and fate, simulation, watersheds, SPARROW, northwestern Gulf of Mexico, South-Central United States

## Abstract

**Abstract:**

SPAtially Referenced Regressions On Watershed attributes (SPARROW) models were developed to estimate nutrient inputs [total nitrogen (TN) and total phosphorus (TP)] to the northwestern part of the Gulf of Mexico from streams in the South-Central United States (U.S.). This area included drainages of the Lower Mississippi, Arkansas-White-Red, and Texas-Gulf hydrologic regions. The models were standardized to reflect nutrient sources and stream conditions during 2002. Model predictions of nutrient loads (mass per time) and yields (mass per area per time) generally were greatest in streams in the eastern part of the region and along reaches near the Texas and Louisiana shoreline. The Mississippi River and Atchafalaya River watersheds, which drain nearly two-thirds of the conterminous U.S., delivered the largest nutrient loads to the Gulf of Mexico, as expected. However, the three largest delivered TN yields were from the Trinity River/Galveston Bay, Calcasieu River, and Aransas River watersheds, while the three largest delivered TP yields were from the Calcasieu River, Mermentau River, and Trinity River/Galveston Bay watersheds. Model output indicated that the three largest sources of nitrogen from the region were atmospheric deposition (42%), commercial fertilizer (20%), and livestock manure (unconfined, 17%). The three largest sources of phosphorus were commercial fertilizer (28%), urban runoff (23%), and livestock manure (confined and unconfined, 23%).

## Introduction

Overabundance of nutrients from freshwater inputs to the northwestern Gulf of Mexico and its coastline continues to be a major cause for concern. The most prominent and well documented issue is hypoxia and degradation of aquatic resources along the inner continental shelf of the Gulf of Mexico off the coasts of Louisiana and Texas. The summer 2008 Gulf hypoxic zone, one of the three largest in size since 1985, encompassed approximately 21,000 km^2^ (Louisiana Universities Marine Consortium, 2010). One of the principal causes for the increasing size of the Gulf hypoxic zone is considered to be the increasing supply of nitrogen (N) and phosphorus (P), particularly nitrate from agricultural sources, delivered to the Gulf each year (Rabalais *et al.*, 1996; Burkart and James, 1999). The availability of nutrients within Gulf waters stimulates excessive phytoplankton growth, which depletes dissolved oxygen (DO) in the bottom water as it dies and decays. These low-DO conditions can be detrimental to fish and other marine life at or near the bottom waters.

The formation and persistence of the Gulf hypoxic zone is largely caused by the discharge of nutrients from the Mississippi-Atchafalaya River basin (MARB) and water column stratification from MARB freshwater inflows (Rabalais *et al.*, 1996; Goolsby and Battaglin, 2000; USEPA, 2010). A goal has been established by a consortium of federal, state, and local partners to reduce the five-year running average areal extent of the Gulf hypoxic zone to about 5,000 km^2^ by 2015 (Mississippi River/Gulf of Mexico Watershed Nutrient Task Force, 2008). Water resource managers, who are charged with development of reduction strategies, have need to prioritize watersheds within the MARB for nutrient load reduction efforts to achieve the Gulf hypoxia reduction goal in the most cost-effective manner. Information needed for prioritization includes determining which watersheds in the MARB deliver the highest nutrient loadings to the Gulf, and what are the primary nutrient sources in those watersheds. Several studies exist in which MARB watersheds are ordered as to their relative contribution to the total nutrient load into the Gulf. For example, Goolsby *et al.* (1999) and Turner and Rabalais (2004) used data from the U.S. Geological Survey (USGS) National Stream Quality Accounting Network (NASQAN) to estimate nutrient loads from the major watersheds of the MARB. Alexander *et al.* (2008) and Robertson *et al.* (2009) used model simulations to estimate nutrient loadings from states and watersheds in the MARB. In most cases, the Upper Mississippi River drainage area, which accounts for about 75% of the MARB drainage area and includes the Missouri, Ohio, and Upper Mississippi River main-stem watersheds, delivers about 80% of the total nitrogen (TN) and about 74% of the total phosphorus (TP) load to the Gulf (Robertson *et al.*, 2009).

It is reasonable to expect that if the majority of the overall nutrient load from the MARB delivered to the Gulf originates from the Upper Mississippi River drainage area, then this part of the MARB would also be prioritized with respect to mitigation activities. For example, the Mississippi River Basin Healthy Watersheds Initiative (MRBI) of the U.S. Department of Agriculture, Natural Resources Conservation Service (USDA-NRCS), was established in 2010 to redirect existing USDA funding to the 12 states identified as top contributors to the overall nutrient load from the MARB to the Gulf (USDA-NRCS, 2010). Forty-one watersheds were selected in the MARB to receive funding to install practices designed to reduce nutrient loads from streams that drain agricultural areas. Of the 41 watersheds selected, 26 were located in the Upper Mississippi River drainage area. Although the Lower Mississippi River drainage area, which includes the Arkansas, White, Red, Yazoo, and Atchafalaya River watersheds, delivers only about a quarter of the TN and TP load to the Gulf, it is still important to understand the Lower Mississippi River's influence on Gulf hypoxia. In addition, nutrient-related issues within the Lower Mississippi River drainage area are locally important to state water resource managers tasked with development of nutrient criteria, total maximum daily loads, and nutrient reduction strategies relative to their state.

Although hypoxia along the inner continental shelf of the Gulf is of national significance, other nutrient-related issues such as localized hypoxia and harmful algal blooms in bays and estuaries along the coasts of Louisiana and Texas in the northwestern Gulf are also becoming more prevalent. Based on work by Bricker *et al.* (2007; see also NOAA, 2010) as part of the National Estuarine Eutrophication Assessment, the influence of excessive nutrients is considered moderate in the Upper Laguna Madre, Corpus Christi Bay, San Antonio River Bay, and Matagorda Bay along the Texas coast and is considered high in the Barataria Bay along the Louisiana coast. Their assessment was not based on a direct measure of nutrient loads to each bay, but rather a measure of “symptoms” of high nutrient loads such as high chlorophyll *a*, low DO, and diminished estuary flushing capacity. Similarly, Clement *et al.* (2001) reported that Calcasieu Lake, Galveston Bay, San Antonio Bay, Corpus Christi Bay, and Laguna Madre had some of the highest levels of eutrophication among all the bays of the Gulf. Thronson and Quigg (2008) reported fish kills in bays along the Texas coast since 1951. Galveston Bay and Matagorda Bay had the largest number of fish kill events and total number of fish killed during their period of study due to low DO. They concluded that the low DO was caused by physical conditions of the bays (temperature, altered hydrology, salinity) and increased algal blooms (toxic and nontoxic) exacerbated by increased inputs of nutrients from upstream watersheds. In Louisiana, nutrient-rich waters from the Mississippi River and adjacent Louisiana drainages have caused changes in phytoplankton species composition in the Lake Pontchartrain basin (Mize and Demcheck, 2009) and caused episodic increases in noxious and harmful algal blooms in Lake Pontchartrain (Dortch *et al.*, 2001) and Barataria Bay (Dortch *et al.*, 1999, 2001). As was discussed for the Lower Mississippi River drainage area, it is important to water resource managers in Louisiana and Texas to quantify nutrient loads delivered to each of the bays and estuaries along their respective coastlines, to determine from where these loads originate in the upstream watersheds, and to determine the primary sources of the nutrient loads.

**FIGURE 1:**
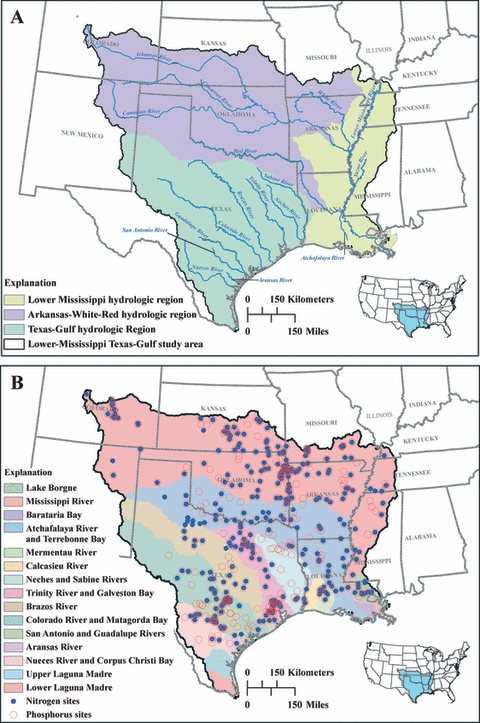
Lower Mississippi Texas-Gulf Region With (A) Hydrologic Region Boundaries and (B) Watershed Boundaries With Calibration Sites Used in the Total Nitrogen and Total Phosphorus SPARROW Models.

SPAtially Referenced Regressions On Watershed attributes (SPARROW) models were developed to assess the sources and delivery of TN and TP from streams in the South-Central United States (U.S.). This area includes the Lower Mississippi, Arkansas-White-Red, and Texas-Gulf hydrologic regions (Figure 1A) (hydrologic unit codes 08, 11, and 12, respectively, as described in Seaber *et al.*, 1987), hereafter referred to as the Lower Mississippi Texas-Gulf (LMTG) region, which drain to coastal waters along the northwestern part of the Gulf of Mexico. Development of these models for the LMTG region was part of regional assessments conducted by the USGS National Water-Quality Assessment (NAWQA) Program to understand water-quality conditions and trends in eight major river basins of the U.S. [Hamilton *et al.*, 2005; see also previous nutrient-related report for the LMTG region by Rebich and Demcheck (2007)]. These models are included with other SPARROW models developed by the NAWQA Program for major regional drainages of the U.S. and reported in this Featured Collection (see Preston *et al.*, this issue). The regional SPARROW models represent an update to national SPARROW models (Smith and Alexander, 2000; Alexander *et al.*, 2008) in that they were based on more calibration sites including those on smaller streams, they were calibrated with more recent data (standardized to the base year of 2002), and they incorporated variables previously unavailable in the national models such as point source data. In addition, models developed for the LMTG region border regional SPARROW models developed for the southeastern U.S. (Hoos and McMahon, 2009; García *et al.*, this issue). Our models coupled with the southeastern SPARROW models provide a complete picture of nutrient loadings and sources from all major watersheds that drain to the Gulf.

Nutrient sources relevant to land-use and landscape conditions in the LMTG region were represented in the models, as well as aquatic and terrestrial processes that influence nutrient transport and delivery. Model simulations presented here could provide baseline information to assist with the development of water management plans, both at the national level in terms of Lower Mississippi River nutrient inputs to the Gulf, and at the local level with respect to individual watersheds draining to a particular bay or estuary along the Louisiana and Texas coasts. This article documents the TN and TP SPARROW models developed for the LMTG region, and presents selected applications of the models such as summarizing load and yield estimates from the entire region, identifying major sources of N and P, and identifying major contributing watersheds based on delivered loads and yields. In addition, this article presents an example of how model output can be used on a local level to identify areas of a coastal watershed where delivered nutrient yields are elevated and to identify the primary sources of nutrients in those areas.

## Methods

The LMTG region encompasses all or parts of 11 states in the South-Central U.S. and includes rivers such as the Lower Mississippi, Yazoo, Canadian, Cimarron, Arkansas, White, Red, Sabine, Neches, Trinity, Brazos, Colorado, San Antonio, and Nueces (Figure 1A). The western part of the LMTG region is fairly rural with few large cities; the eastern part is also rural with respect to land area but is more populous, containing 2 of the top 10 metropolitan centers in the U.S. (Dallas-Ft. Worth and Houston-Galveston, U.S. Census Bureau, 2001). Temperature gradients do not vary considerably throughout the LMTG region, but rainfall patterns do vary, with fairly arid conditions in the western part and humid subtropical conditions in the eastern part that result in frequent annual inputs of moisture from the Gulf of Mexico (Owenby *et al.*, 2001). Thus, land-use patterns typically reflect rainfall and water availability in that the more arid part of the region in the west is home to large tracts of pasture and rangeland while the eastern part is well-known for large areas of row-crop production. Implications are that streamflow and nutrient loadings will be larger in the eastern part than in the western part of the LMTG region due to rainfall and land-use patterns.

Estimates of TN and TP from the LMTG SPARROW models represent nutrient contributions from the fluvial drainages that enter bays and estuaries along the Louisiana and Texas coasts or that discharge directly to the Gulf of Mexico. The TN and TP model estimates do not reflect the effects of processes that occur within the waters of any particular bay or estuary. Implications are that load estimates presented here may be relevant to water-quality and eutrophication issues in bays and estuaries (e.g., localized hypoxia and harmful algal blooms), but the load estimates from a bay or estuarine watershed may not be relevant to hypoxia on the inner continental shelf of the northern Gulf of Mexico. For ease of use, however, the estimates of TN and TP are summarized graphically for the major watersheds of the LMTG region, and it is inferred that each drains to the northwestern Gulf of Mexico. The watersheds and their associated names are identified according to the NOAA Coastal Assessment Framework (Figure 1B) (NOAA, 2007) for central-western Gulf of Mexico estuaries, except for the following selected watersheds, which were combined to simplify the presentation: (1) the Lake Borgne watershed includes reaches from the Breton/Chandeleur Sound watershed, (2) the Atchafalaya River/Terrebonne Bay watershed is a combination of the Atchafalaya/Vermillion Bays and the Terrebonne/Timbalier Bays watersheds, and (3) the Upper Laguna Madre watershed includes reaches from the Palo Blanco River watershed. In addition, some of the watershed names did not reflect important rivers or tributaries as part of their NOAA-assigned name and were revised to include those important rivers. For example, the Galveston Bay watershed was revised to be the Trinity River/Galveston Bay watershed (Figure 1B).

SPARROW models use a hybrid statistical and process-based approach that relates nutrient loads (or mass) to upstream sources, landscape characteristics that influence nutrient transport, and instream loss (Smith *et al.*, 1997; Smith and Alexander, 2000; Preston *et al.*, 2009). The input datasets for the model are spatially referenced to a digital stream reach network, and in this case, the enhanced Reach File 1 network, version 2 (eRF1_2), was used to estimate basin characteristics for each reach such as drainage area, stream velocity, slope, and flow (USEPA, 1996; Brakebill *et al.*, this issue). This reach network serves to relate upstream and downstream loads, and for any specific reach, the SPARROW model estimates loads totaled for all upstream reaches as well as loads generated for a particular reach. Catchments were generated for each eRF1_2 reach, and these catchments were used to allocate spatial data for nutrient source and landscape and aquatic characteristics data to each reach (Wieczorek and Lamotte, 2011; unless otherwise noted all spatial data in this article are from this source).

The SPARROW model uses nonlinear least squares regression during the calibration process, in which nutrient sources are weighted by estimates of loss due to overland and instream processing (Preston *et al.*, 2009). Load estimates at sampled locations are the “dependent” variables in the SPARROW model during the calibration process; and source, landscape characteristic, and instream loss terms are the “independent” variables (landscape characteristic terms are, hereafter, referred to as land-to-water delivery terms). Source terms are included in SPARROW models to help explain variability in loads leaving stream reaches. Land-to-water delivery terms are included to determine their significance on the delivery of nutrients from the land surface to LMTG stream reaches. Instream loss terms are included to represent the amount of nutrients lost as upstream sources are related to downstream loads. Spatial referencing is retained for all input terms in the model so that estimated loads can be interpreted in a spatial context. Further information about the mathematical form of the SPARROW model can be found in Schwarz *et al.* (2006) and in the Supporting Information for this article.

Total nitrogen and TP loads (dependent variable) were estimated using a software package called Fluxmaster, which uses an adjusted maximum likelihood approach as described in Schwarz *et al.* (2006). TN and TP loads were determined with log-linear water-quality regression models that relate the logarithm of constituent concentration to the logarithm of daily flow. The regression models compensate for trends in the data and seasonality (expressed using trigonometric functions of the fraction of the year). The mean annual load for each sampling location is standardized to the 2002 base year, which means that the estimate of the mean nutrient load is one that would have occurred in 2002 if mean annual flow conditions from a much longer period of time had prevailed (in this case, 1980-2002). The standardization process can also be referred to as “detrending,” in that the time series of nutrient load estimates at a particular site is “pivoted” on the base year. This process removes trends in load datasets at individual sites, if they exist, so that load estimates at all calibration sites are comparable prior to the calibration process. Source and land-to-water delivery data were also summarized for the 2002 base year (further explanation about the concept of the base year is given in Preston *et al.*, 2009).

Total nitrogen and TP concentration data used to estimate loads were acquired from the USGS, USEPA, and databases from the states of Mississippi, Louisiana, Oklahoma, Texas, Arkansas, and Kansas. TN and TP concentration data from the various federal, state, and local databases were assumed to be of similar quality although sampling protocols and quality assurance procedures likely differed. Sites selected for model calibration were screened using criteria related to the type and amount of water-quality data available at each site. Selected sites were then matched with nearby streamflow gaging stations, and mean daily flow data used for load estimation were acquired from USGS and selected U.S. Army Corps of Engineers (USACE) gaging stations. Flow record was considered usable for load estimation if inclusive of the 2002 base year. A complete description of the screening and collocation process is available in the Supporting Information as well as in Saad *et al.* (this issue). Once the screening process was completed, there were 344 calibration sites available for the TN model and 442 calibration sites available for the TP model (Figure 1B). The median drainage size for both sets of calibration sites was about 700 km^2^. In comparison, the national model developed by Alexander *et al.* (2008) was calibrated with 425 sites across the U.S., of which the median drainage area was about 10,500 km^2^ and only 68 were located within the LMTG region.

Selection of source, land-to-water delivery, and loss terms (independent variables) considered for LMTG SPARROW models was guided by: (1) review of terms selected for the national SPARROW model (Alexander *et al.*, 2008) and for the southeastern U.S. regional SPARROW TN model (hereafter referred to as SE-TN model, Hoos and McMahon, 2009), and (2) a review of sources and potential delivery mechanisms identified in local studies (e.g., van Metre and Reutter, 1995; Davis and Bell, 1998; Ging, 1999; Coupe, 2002; Haggard *et al.*, 2003; Demcheck *et al.*, 2004; Tortorelli, 2008). Urban sources considered in both the TN and TP models included load estimates for municipal and industrial point sources, urban runoff based on residential land-use classes, and impervious surface area. Point source data for the year 2002 were used directly, if available, from the USEPA Permit Compliance System (PCS) (USEPA, 2009), or were estimated using methods documented by Maupin and Ivahnenko (this issue). Agricultural sources considered in both models were fertilizer applied to agricultural lands and livestock manure from confined and unconfined animal feeding operations. N and P fertilizer and livestock manure sources were further refined on the basis of crop type. All agricultural source datasets were based on county-level estimates for each state in the LMTG region. Other sources considered only for the TN model included wet deposition of total inorganic nitrogen (TIN), which is the combination of wet deposition of ammonia-N and nitrate-N (hereafter referred to as atmospheric deposition, and similarly detrended to 2002 as were load estimates at sampled locations), and fixation of N by selected crops such as soybean, alfalfa, and hay. Other sources considered only for the TP model were P attached to suspended material from in-channel erosion and background sources of P (forest, wetlands, scrub, and barren land-use categories). In addition, the LMTG region includes only the lower portion of the Mississippi River basin; therefore, a TN and TP load for the Upper Mississippi River basin was assigned as a “source” to the uppermost Mississippi River reach in the LMTG region as a boundary condition for each model. The Upper Mississippi load estimates were calculated by summing load data for 2002 for the Upper Mississippi River main stem, the Missouri River, and the Ohio River as published from the USGS NASQAN program (Aulenbach *et al.*, 2007).

Land-to-water delivery terms considered for both models were precipitation (average for 2002 and 30-year average), soil permeability, channel slope, overland flow in excess of infiltration, overland flow in excess of saturation, drainage density, surficial geology classifications, bedrock geology classifications, hydrologic landscape regions, groundwater recharge, and estimated area of irrigated agricultural lands. Land-to-water delivery terms considered only for the TP model were estimated area of dams not included in the eRF1_2 reach network, average clay content, average silt content, and soil erodibility factor (*K*-factor from the Universal Soil Loss Equation).

Nutrient removal, or loss, in streams was modeled in SPARROW according to a first-order decay process, in which the fraction of contaminant removed in a given stream reach is estimated using an exponential function of an instream loss rate and travel time in the stream reach (Preston and Brakebill, 1999; Schwarz *et al.*, 2006). In this approach, the loss simulated by SPARROW is a consequence of the combination of all biological or chemical processes that may contribute to nutrient loss in streams. Individual loss processes such as denitrification in the TN model were not considered separately. Loss associated with stream transport can vary by stream size (Alexander *et al.*, 2000; Schwarz *et al.*, 2006); therefore, loss in both the TN and TP models was estimated for LMTG streams by considering three stream size classes defined by flow percentiles as: (1) streams with flows ≤1.4 m^3^/s (roughly 10% of all average annual flows for stream reaches in the region), (2) streams with flows >1.4 and ≤28 m^3^/s (28 m^3^/s or less represents roughly 75% of all annual flows for stream reaches in the region), and (3) streams with flows >28 m^3^/s. Loss in reservoirs was also modeled as a first-order decay process, and expressed as an apparent settling velocity (or mass transfer coefficient) in units of length per time. Reservoir loss is estimated as a function of the ratio of outflow discharge and surface area of the reservoir, and it represents the net effect of processes that remove nutrients from the water column to reservoir sediments and processes that add nutrients back to the water column (e.g., mineralization, dissolution, and resuspension) (Schwarz *et al.*, 2006).

Coefficients for each considered term were computed during the calibration process (nonlinear least squares regression), and the coefficients were evaluated for statistical significance. The final calibrated SPARROW models were selected based on assessment of significance level (α = 0.05) and interpretability of each source, land-to-water delivery, and loss term. Model coefficients, their standard errors and significance levels (*p*-values), and 90th percentile confidence limits are presented in this article for each model. Confidence limits for the coefficients were computed using a *t*-distribution with *N*− *k* degrees of freedom, where *N* is the number of monitoring locations, and *k* is the number of coefficients estimated in the model. The robustness of the coefficients of the final TN and TP models were examined using nonparametric resampled bootstrapping procedures with 200 iterations. The bootstrapping procedures produce a mean value for the coefficients in each model (see Schwarz *et al.*, 2006, for more information about bootstrapping procedures).

Model performance, or goodness of fit, was evaluated on the basis of root mean square error, coefficients of determination (*R*^2^), and magnitude and spatial distribution of residuals. A residual is an expression of the difference between the measured loads used for calibration and the model-estimated loads. For this article, residuals are standardized to have zero mean and unit variance and are referred to as studentized residuals (see Schwarz *et al.*, 2006, for further explanation and derivation of a studentized residual). Spatial distribution of residuals was evaluated for regional biases including land use, geology, hydrology, and other possible explanatory considerations, each of which led to additional model runs for evaluation as a possible source or land-to-water delivery term in the models. Further explanation of statistical values that were used to evaluate model performance can be found in the Supporting Information. The final model was selected on the basis of (1) lowest root mean square error, (2) highest yield *R*^2^ (yield is calculated as load divided by contributing drainage area, therefore, yield *R*^2^ is the coefficient of determination adjusted for scaling effects due to drainage area), (3) lowest residual magnitudes, and (4) residuals with the lowest degree of spatial bias based on visual inspection of mapped residuals.

Model output were mean annual predictions of nutrient mass for all stream reaches in the LMTG region and include the load (mass per time), yield (mass per unit area per time), concentration (mass per unit water volume), and source-share contributions (percentage of the load for each source). Stream load and yield were reported for three spatial domains: (1) total drainage area upstream of an individual reach outlet, (2) the incremental reach drainage area, which is mass delivered to the downstream end of an individual reach exclusively from sources in the catchment that drain directly to the reach without passing through another reach (e.g., the incremental drainage area), and (3) the amount of mass delivered from an incremental or total drainage area from an individual reach to a downstream water body, for example, estuary, reservoir. Their corresponding metrics are hereafter referred to as the “total,”“incremental,” and “delivered” load or yield, respectively. These metrics provide management-relevant information about the sources and fate of nutrients from local to regional spatial scales. The delivered load or yield was calculated by multiplying the total or incremental value of a stream reach by the SPARROW estimate of the “delivery fraction,” which quantifies the proportion of the nutrient load that is delivered to downstream waters without any removal by natural attenuation processes. (Note: definitions presented here are modified from USGS, 2010).

Model output presented in this article includes maps of incremental and delivered incremental TN and TP yield estimates generated for the entire LMTG region and for each individual watershed. Statistical information that summarizes incremental yields and source shares for the entire LMTG region are tabled, and maps are included that present primary source shares for each incremental drainage area. In addition, this article presents delivered load and yield estimates that were accumulated for each of the 15 LMTG watersheds (tabled). These estimates describe the cumulative mass generated in a watershed from all stream reaches that terminate at the watershed outlet. The accumulated delivered load and yield estimates were produced for these watersheds using a parametric bootstrapping approach with 200 model iterations, so the estimates include corresponding standard errors and 90th percentile confidence limits. More details are available in the Supporting Information describing computations used to accumulate delivered loads and yields by watershed.

## Results and Discussion

### Model Calibration Results

Source, land-to-water delivery, and loss terms for the final TN and TP SPARROW models for the LMTG region are presented in Table 1. The final TN model included six source terms, which were atmospheric deposition, industrial and municipal point sources, urban runoff from residential land-use classes, livestock manure from confined and unconfined animal feeding operations (separate terms), and fertilizer applied to crops; two land-to-water delivery terms, which were overland flow in excess of infiltration and 30-year average precipitation; two instream loss terms, which were for streams with average streamflows ≤1.4 m^3^/s and streams with average streamflows >1.4 and ≤28 m^3^/s; and one reservoir loss term. All final terms in the TN model were highly significant (*p* < 0.01).

**TABLE 1:** Model statistics for the total nitrogen and total phosphorus SPARROW models developed for the Lower Mississippi Texas-Gulf region

			90th confidence interval for mean coefficient				
						
Predictor variable description	Model coefficient units	Mean coefficient	Lower	Upper	Standard error of mean coefficent	Probability level (p-value)	Nonparametric bootstrap estimate of coefficient (mean)
**Total nitrogen**							
**Sources**							
Atmospheric deposition^1^ (kg/yr)	dimensionless	0.216	0.148	0.284	0.041	<0.001	0.208
Point sources^2^ (kg/yr)	dimensionless	1.39	0.943	1.84	0.271	<0.001	1.35
Urban runoff^3^ (km^2^)	kg/km^2^/yr	609	358	860	152	<0.001	577
Livestock manure, confined^4^ (kg/yr)	dimensionless	0.169	0.079	0.260	0.055	0.001	0.166
Livestock manure, unconfined^5^ (kg/yr)	dimensionless	0.075	0.029	0.122	0.028	0.004	0.075
Farm fertilizer^6^ (kg/yr)	dimensionless	0.061	0.040	0.083	0.013	<0.001	0.059
**Land-to-water delivery**							
Infiltration excess overland flow^7^ (%)	dimensionless	0.027	0.018	0.036	0.006	<0.001	0.027
Precipitation^8^ (ln(mm))	per mm	1.65	1.29	2.03	0.224	<0.001	1.73
**Aquatic loss**							
Streams^9^, = 1.4 m^3^/s (days)	per day	0.365	0.230	0.499	0.082	<0.001	0.329
Streams^10^, 1.4 < Q = 28 m^3^/s (days)	per day	0.079	0.044	0.114	0.021	<0.001	0.078
Reservoirs^11^ (yr/m)	m/yr	12.1	7.56	16.6	2.75	<0.001	11.70
**Model diagnostics**							
Number of observations	344		R^2^ load^12^		0.919		
Mean square error	0.304		R^2^ yield^13^		0.863		
Root mean square error	0.552						
**Total Phosphorus**							
**Sources**							
Point sources^2^ (kg/yr)	dimensionless	1.86	1.18	2.53	0.407	<0.001	1.79
Urban runoff^3^ (km^2^)	kg/km^2^/yr	106	68.6	144	22.8	<0.001	105
Farm fertilizer^6^ (kg/yr)	dimensionless	0.058	0.037	0.079	0.013	<0.001	0.058
Livestock manure^14^ (kg/yr)	dimensionless	0.019	0.011	0.027	0.005	<0.001	0.019
In-channel erosion^15^ (m)	kg/m	0.034	0.011	0.057	0.014	0.008	0.036
Background sources^16^ (km^2^)	kg/km^2^/yr	2.04	0	4.10	1.26	0.050	1.44
**Land-to-water delivery**							
Infiltration excess overland flow^7^ (%)	dimensionless	0.033	0.021	0.045	0.007	<0.001	0.033
Precipitation^8^ (ln(mm))	per mm	2.33	1.80	2.87	0.320	<0.001	2.34
Soil erodibility^17^ (dimensionless)	dimensionless	8.83	6.32	11.3	1.52	<0.001	8.74
**Aquatic loss**							
Streams^9^, = 1.4 m^3^/s (days)	per day	0.250	0.144	0.364	0.067	<0.001	0.260
Reservoirs^11^ (yr/m)	m/yr	8.67	4.35	13.0	2.62	0.001	8.03
**Model diagnostics**							
Number of observations	442		R^2^ load^12^		0.878		
Mean square error	0.552		R^2^ yield^13^		0.798		
Root mean square error	0.743						

Note: SPARROW, SPAtially Referenced Regressions On Watershed attributes.

1 Average annual wet deposition of inorganic nitrogen (ammonia and nitrate), detrended for 2002.

2 Surface-water discharges from permitted wastewater discharge, municipal and industrial sources, 2002.

3 Developed land use classes (high, medium, low, and open) used as surrogate for urban runoff, 2001.

4 Livestock manure from confined animal feeding operations, 2002.

5 Livestock manure from unconfined animal feeding operations, 2002.

6 Commercial fertilizer applied to agricultural land, 2002.

7 Based on work by Beven and Kirkby (1979), in percent of streamflow for reach catchment.

8 30-year average precipitation, 1971-2000, natural log of mm.

9 Loss of nitrogen and/or phosphorus in reaches with mean daily discharges less than or equal to 1.4 m3/s.

10 Loss of nitrogen in reaches with mean daily discharges greater than 1.4 and less than or equal to 28 m3/s.

11 Loss of nitrogen and/or phosphorus through reservoirs is expressed as the inverse areal hydraulic load.

12 Coefficient of determination of the log-transformed load estimate.

13 Coefficient of determination of the log-transformed yield estimate.

14 Livestock manure combined from confined and unconfined animal feeding operations, 2002.

15 Surrogate for phosphorus attached to sediment from in-channel erosion, expressed as reach channel length.

Reaches whose mean daily discharges were less than or equal to 1.4 m^3^/sec were not statistically significant.

16 Surrogate for background sources of phosphorus based on area of forest, wetland, barren, and scrub land, 2001.

17 K-factor from Universal Soil Loss Equation.

The final TP model included six source terms, which were industrial and municipal point sources, urban runoff from residential land-use classes, fertilizer applied to crops, livestock manure from confined and unconfined animal feeding operations (combined term), sediment from in-channel erosion, and background sources; three land-to-water delivery terms, which were overland flow in excess of infiltration, 30-year average precipitation, and soil erodibility factor (or *K*-factor); one instream loss term, which was for streams with average streamflows ≤1.4 m^3^/s; and one reservoir loss term. Nearly all final terms in the TP model were significant (*p* ≤ 0.05).

Source, land-to-water delivery, and loss terms used in LMTG models were comparable to other SPARROW models (Smith *et al.*, 1997; Alexander *et al.*, 2008; Hoos and McMahon, 2009) in terms of broad categories such as urban, atmospheric deposition, applied fertilizer, livestock manure, and background sources. However, there were differences between terms used in LMTG and other SPARROW models due to the need to specify and refine the models relevant to the LMTG region (which helped to explain variability observed in nutrient loadings and to reduce errors) and due to use of datasets previously unavailable to other models. For example, LMTG models utilized point source data from the USEPA PCS database and residential land-use classes from the 2001 National Land Cover Database (NLCD) to account for urban sources, whereas Alexander *et al.* (2008) used population as a surrogate to represent urban sources in the national model. Other departures included agricultural fertilizer sources, which were combined for all crop types in the LMTG models and in previous national models by Smith *et al.* (1997), but were split according to crop type for national models developed by Alexander *et al.* (2008). Attempts to account for agricultural fertilizer by crop type for the LMTG models were unsuccessful due to the fact that several crops were never statistically significant in LMTG model runs. Also, livestock manure from confined and unconfined animal feeding operations was treated as a combined source in the LMTG-TP model similar to other SPARROW models. However, livestock manure from confined and unconfined animal feeding operations remained as separate source terms in the TN model because both terms were statistically significant during the calibration process providing better refinement of the TN model for the LMTG region.

Model coefficients produced by the nonlinear least squares regression methods were robust and compared well to those produced by the nonparametric bootstrap methods, and in most cases, were within 5% of each other. Exceptions included one instream loss term in the TN model (difference in the coefficients from the two methods was about 10%) and the background source term in the TP model (difference in the coefficients from the two methods was about 34%).

*R*^2^ values were 0.92 and 0.88, and the yield *R*^2^ values were 0.86 and 0.80 for the TN and TP models, respectively, indicating that the assembled set of predictor variables used for the TN model explained variability in observed loads slightly better than did the predictor variables used in the TP model. Model uncertainty associated with load predictions for any given reach was lower for the TN model than the TP model as indicated by the root mean square errors, which were 0.55 and 0.74, respectively.

**FIGURE 2:**
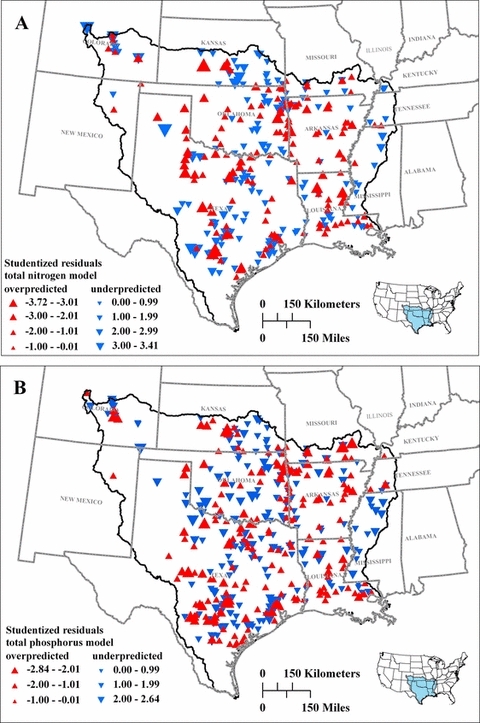
(A) Total Nitrogen and (B) Total Phosphorus Studentized Residuals From Lower Mississippi Texas-Gulf SPARROW Models. A residual is an expression of the difference between the measured loads used for calibration and the model-estimated loads. For this article, residuals are standardized to have zero mean and unit variance and are referred to as studentized residuals.

Studentized residuals of the TN and TP models for the LMTG region are plotted in Figure 2. Positive residuals indicate areas where the model underpredicts loads, and negative residuals indicate areas where the model overpredicts loads. Good model fit is indicated by: (1) residuals that are distributed randomly with no spatial patterns that could indicate a particular bias in the model for a specific geographic location, and (2) few residual “outliers,” with an outlier being defined for this article as <−3 or >3. For the TN model residuals (Figure 2A), the model fits particularly well with no obvious spatial patterns. There were six TN residuals that were <−3 or >3 indicating possible outliers. The water quality and streamflow datasets at those sites were further investigated to ensure that there were no errors within each. No substantive reason to delete any of the six from calibration was found, and the conclusion was that the model simply did not fit well at these locations. For the most part, there were also no apparent spatial patterns in the TP residuals except for Mississippi where the model underpredicts TP loads. In addition, there were no TP residual magnitudes <−3 or >3.

### Nitrogen and Phosphorus Sources

The present application of SPARROW includes nonconservative transport and mass-balance constraints. Given a specification of nutrient sources, the model estimates nutrient delivery from these sources to streams in relation to the land-to-water delivery terms specified in the model, which in the case of the LMTG models, included climate, infiltration excess overland flow, and soil erodibility (TP model only). Coefficients estimated for each source are expressed as either a percentage (fraction) for the mass variables delivered to the streams, or a unit area load to streams (kg/km^2^) for the land-use variables. Source coefficients account for losses (or gains) in the delivery of mass to all stream reaches throughout the model domain. Spatially variable land-to-water delivery factors such as climate and infiltration may account for additional gains or losses in delivery of mass to streams in areas where those variables affect loading.

Urban sources for both LMTG models included point sources and urban runoff. Point source loads were input as a mass (in kg) to both models and response variables were expressed in the same units. Model coefficients for point sources were expected to be near 1 (dimensionless), which infers that 100% of the input is delivered to the stream, as it is assumed that point sources discharge directly to receiving streams and their loads are unaffected by land-to-water delivery factors. However, coefficients for point sources in both the TN and TP models were >1 (Table 1), indicating that during the calibration phase (comparison of estimated stream loads to measured loads) it was necessary to increase point source contributions above what was expected in order to meet the mass-balance constraints of the model. A possible explanation is that point source data used for these models do not fully account for, and thus underestimate, point source contributions in the LMTG region. In addition, the influence of septic systems and unsewered communities are unaccounted for in the model because data describing these sources are generally unavailable. For these reasons, the coefficients for point sources in the LMTG models being >1 are considered a limitation, in that the models indicate more mass of nutrients from point sources being delivered from streams than mass introduced to streams in the LMTG region.

Urban nonpoint runoff is included in the model as an area term (in km^2^) expressed as a summation of land area for low, medium, high, and open space developed land-use categories from the 2001 NLCD. Developed land, therefore, serves as a surrogate measure of various diffuse urban sources in the model. These sources may include nutrient runoff from impervious surfaces and inflows from surface and groundwaters in urbanized catchments related to such sources as fertilizers, septic systems, sewage collection system leaks, sewage collection system overflows and bypasses, combined sewer overflows (where they exist), and atmospheric deposition from vehicle emissions. The model coefficient for urban runoff, expressed as kg/km^2^/year, from the TN model is about six times higher than the coefficient from the TP model (609 and 106 kg/km^2^, respectively, as shown in Table 1). Both coefficients agreed fairly well with published ranges, such as those found in Beaulac and Reckhow (1982), who completed a literature search to quantify ranges of N and P export rates for selected land-use categories. From their research, N export coefficients for urban nonpoint runoff typically ranged between about 400 and 1,200 kg/km^2^/year and were about six times higher than P export coefficients, which typically ranged between 60 and 270 kg/km^2^/year.

Agricultural sources in both LMTG models include fertilizer and livestock manure. Fertilizer inputs to the LMTG models are based on county-level fertilizer sales. Therefore, farm fertilizer sales serve as a measure of the location and intensity of farming activities; in addition to providing a direct measure of commercial fertilizer use, the fertilizer source in the LMTG models serves as a surrogate for other nutrient inputs to farms and the net effects of farm practices on nutrient runoff to the extent that they are spatially correlated with fertilizer sales. Therefore, model estimates of fertilizer contributions to streams may potentially reflect additional nutrient inputs to croplands from manure-based fertilizers and N fixation by legumes (e.g., soybeans, alfalfa) and the effects of some farm management practices (e.g., rotations, harvesting, conservation tillage). Nutrient mineralization and immobilization rates in cultivated soils are assumed to be approximately in equilibrium. Nutrients associated with livestock manure reflect contributions from the excreted wastes of *confined* animals, including those in concentrated animal feeding operation, and excreted wastes from *unconfined* animals on farms, pastures, and rangelands. Confined animal wastes include recoverable manure that may be applied to nearby farmlands as well as unrecoverable manure that is lost during the collection, storage, and treatment of the waste.

Coefficients for agricultural sources represent the net mean fraction of the source delivered from the land surface to the stream given the effect of land-to-water delivery losses specified in each model relevant to conditions in the LMTG region. For example, the coefficient for N from fertilizer applied to crops is 0.061 in the TN model (Table 1). In general, setting aside the effects of spatially variable land-to-water terms, this particular coefficient in the TN model indicates that for every kg of N fertilizer that is applied to the landscape, about 0.06 kg (or 6%) is delivered to a nearby stream. The remaining 0.94 kg of applied N fertilizer is removed either at the point of application (plant uptake and harvest) or during the transport process from land surface to the stream. The amount of N from agricultural sources delivered to streams in the LMTG region was as follows: about 6% (0.061 kg/kg) of applied fertilizer, about 17% (0.169 kg/kg) of livestock manure from confined feeding operations, and about 8% (0.075 kg/kg) of livestock manure from unconfined feeding operations (Table 1). These source coefficients can be vastly different from one area of the U.S. to another depending on crop type, soils, land-management practices, and other factors. As an example, rate coefficients for the SE-TN model were about 12% (0.12 kg/kg) for applied fertilizer and about 5% for (0.05 kg/kg) for manure from livestock production (Hoos and McMahon, 2009). The percent of P from agricultural sources delivered to streams in the LMTG region was as follows: about 6% (0.058 kg/kg) of applied fertilizer and about 2% (0.019 kg/kg) of livestock manure from the combination of confined and unconfined animal feeding operations (Table 1).

Atmospheric deposition estimates used as input to SPARROW are based on the use of wet deposition measurements at National Atmospheric Deposition Program (NADP) sites as a surrogate for wet plus dry inorganic N deposition. LMTG-TN model estimates of atmospheric N deposition delivered to streams is expected to account for additional contributions from dry N deposition forms because regional patterns of wet and dry deposition are generally correlated over large areas of the U.S. (Baumgardner *et al.*, 2002; Holland *et al.*, 2005). LMTG-TN model estimates of atmospheric N contributions to streams primarily reflect *regional* atmospheric N sources, given that NADP wet-deposition estimates generally reflect regional N emissions from both agricultural and industrial stationary sources (Elliott *et al.*, 2007). In the LMTG region, atmospheric depositional patterns generally follow ammonia deposition rather than nitrate deposition (Wieczorek and Lamotte, 2011) indicating more of an agricultural influence (fertilizer and livestock manure) on the TN model. Also, local atmospheric N sources, such as those associated with vehicle emissions, are likely to be included in contributions from other modeled N sources, especially urban sources (e.g., developed or impervious land).

About 22% (0.216 kg/kg) of N from atmospheric deposition onto the land surface was delivered to streams in the LMTG region on average; again, setting aside the effects of spatially variable land-to-water delivery terms (Table 1). The atmospheric deposition rate from the LMTG region is about half the rate for streams in the southeastern U.S., which was about 50% (0.5 kg/kg, SE-TN model) (Hoos and McMahon, 2009). Alexander *et al.* (2001) explored atmospheric N flux from watersheds of major estuaries of the U.S. In their work, average atmospheric deposition export rates were only slightly higher for southeastern watersheds than for LMTG watersheds. However, their work is not directly comparable to LMTG and SE-TN SPARROW model coefficient values because they only considered nitrate-N, whereas the SE-TN and LMTG-TN models included a combination of nitrate and ammonia N (or TIN). Climatic factors could be the main reason for the lower delivery rates of atmospheric N from the LMTG-TN model than from the SE-TN model. Rainfall in the LMTG region varies substantially with much lower rainfall in the western part (about 50 cm/year) than in the eastern part of the region (about 140 cm/year), and rainfall patterns in the eastern part of the LMTG region would be fairly comparable to rainfall patterns for the entire region of the SE-TN model.

Stream length (or stream channel) was found to be a significant source of sediment in a recent national application of the SPARROW model (Schwarz, 2008). Stream length was included in the TP model because P bound to mobilized sediment from in-channel erosion is considered a likely source of P in the LMTG region. As P is delivered to the stream directly via point sources, such as industrial and municipal wastewater treatment plants, and indirectly via nonpoint sources, such as urban runoff, agricultural fields, and natural/geologic sources, it is sorbed to suspended sediment during runoff events when stream velocities and availability of channel material are highest. Bound P can be stored in the stream bed as velocities subside and sediment settles, but during subsequent runoff events, P can be transported attached to re-suspended sediment particles and/or desorbed and released back to the water column (Owens and Walling, 2002; van der Perk *et al.*, 2006). Stream length was initially introduced into the TP model using two flow classes – streams with mean daily flows ≤1.4 m^3^/s and streams with mean daily flows >1.4 m^3^/s. The final model included stream length only for streams with mean daily flows >1.4 m^3^/s indicating that streams with smaller flows were less capable of producing and transporting large amounts of sediment from in-channel erosion. P contribution from in-channel erosion was about 0.03 kg/m of stream length on average for the LMTG region (Table 1). Because the TP model represents long-term steady-state conditions, inclusion and statistical significance of stream length as a source of P indicates that P attached to sediment from channel erosion and scouring may be ongoing, and long-term equilibrium with deposition has not yet been attained for medium to large streams in the LMTG region.

Land-use categories comprising the background source of P for the LMTG-TP model were NLCD categories for forests (deciduous, evergreen, and mixed), wetlands (woody and emergent herbaceous), scrub, and barren. Each of these categories was considered a separate source term in previous model runs, but some were not statistically significant. To account for all sources of P in the LMTG region, these land-use categories were combined into a single background source term as presented here, which was statistically significant in the final LMTG-TP model. On average, about 2 kg/km^2^/year of P are delivered to streams in the LMTG region from these background sources (Table 1). By comparison, P from background sources from the LMTG model were lower than P export rates for forests which typically range between 10 and 30 kg/km^2^/year as reported by Beaulac and Reckhow (1982). Background source term coefficients in the national SPARROW model were as follows: forest land, 16.7 kg/km^2^/year; barren/transitional land, 135 kg/km^2^/year; and shrub land, 22.7 kg/km^2^/year (Alexander *et al.*, 2008). A possible explanation for the discrepancy between the export rate for the background source term from the LMTG-TP model and literature is that the LMTG-TP model also included stream length as a separate source, which represented P attached to sediment from in-channel erosion. This sediment-P term in the TP model could also be accounting for a portion of the exported P from those areas in the LMTG region categorized as background. Export rates reported in Beaulac and Reckhow (1982) and from the national SPARROW model likely reflect a combination of both dissolved and sediment/particulate P from similar areas.

### Land-to-Water Delivery

Land-to-water delivery variables common to both models were precipitation and overland flow in excess of infiltration, which were highly significant in both models (*p*-values <0.01 as shown in Table 1). Precipitation is expressed as the natural log of the 30-year average from 1971 to 2000 in millimeters. Precipitation affects the amount and rate of surface water runoff available to transport TN and TP to streams in the LMTG region. With much higher precipitation in the eastern part of the LMTG region, it is expected that TN and TP loadings in streams in the eastern part of the region would be higher than those in the western part. Overland flow in excess of infiltration is a term based on the work of Beven and Kirkby (1979) and is estimated from storm hydrographs where precipitation rates exceed infiltration rates, so in a sense, represents runoff potential for the LMTG region. Overland flow in excess of infiltration is highest and a major part of storm hydrographs for landscapes that are disturbed or poorly vegetated in subhumid or semiarid climates. The overland flow in excess of infiltration term is expressed in percent of streamflow in the models with higher percentages (30-70%) in the western and coastal parts of the region and lower percentages (5-20%) in the eastern parts. Coefficients for precipitation and overland flow in excess of infiltration in both models are positive indicating that each term enhances transport of nutrients to nearby streams.

The LMTG-TP model also included soil erodibility as a land-to-water delivery term, and soil erodibility was highly significant (Table 1). The soil erodibility term in the TP model is the Soil Erodibility Factor, or *K*-factor, from the Universal Soil Loss Equation made available from the State Soil Geographic (STATSGO) database (USDA, 1994). Soil erodibility factor represents the susceptibility of soil particles to detachment and transport in runoff. The higher the value, the more susceptible a particular soil is to both detachment and transport. More than 50% of the LMTG region has soil erodibility values >0.25. The coefficient for soil erodibility in the TP model is positive indicating that soil erodibility enhances TP movement into nearby streams from the landscape.

### Instream Loss

For the TN model, the instream loss coefficient for stream reaches with mean daily flows ≤1.4 m^3^/s was about 0.37 day^−1^ (Table 1). The instream loss coefficient for stream reaches with mean daily flows >1.4 and ≤28 m^3^/s was about 0.08 day^−1^ (Table 1). Both decay terms were highly significant (*p* < 0.001) and were relatively consistent with other regional and national SPARROW models (Preston and Brakebill, 1999; Alexander *et al.*, 2008; Hoos and McMahon, 2009). Instream loss of N for stream reaches with mean daily flows >28 m^3^/s was not statistically significant suggesting that instream processing of N for larger stream reaches was minimal. These findings support the understanding that N decline in streams has an inverse relation with increases in water depth and stream size and that N removal processes such as denitrification and settling of particulate matter are more inhibited in large streams (Alexander *et al.*, 2008). The loss of N in reservoirs was 12.1 m/year (Table 1), which compared well to the SE-TN SPARROW model which had a reservoir loss rate of 13.1 m/year (Hoos and McMahon, 2009).

Instream loss of P was initially modeled using the same stream class breaks (based on flow classes) as were used for the TN model. However, for all preliminary runs of the TP model, coefficients of instream loss of P for stream reaches with mean daily flows >1.4 m^3^/s were consistently insignificant (*p*-values >0.05). For the final LMTG-TP model, the only instream P loss term that was included was for stream reaches with mean daily flows ≤1.4 m^3^/s, and for these stream reaches, the P loss rate was 0.25 day^−1^ (Table 1). It should be noted that the same stream size classification (mean daily flows of 1.4 m^3^/s) was used for both the stream length term as a source (sediment from in-channel erosion) and here as a stream loss term. For stream reaches with mean daily flows >1.4 m^3^/s, stream length was an important source and loss was minimal. These results imply that, for streams whose mean daily flows are >1.4 m^3^/s, P bound to sediment continues to be transported downstream with minimal loss even with settling and re-suspension phases considered. The loss of P in reservoirs in the LMTG region was 8.67 m/year (Table 1), which is similar to the reservoir loss rate for N.

### Distribution of Yields and Sources Throughout the LMTG Region

**FIGURE 3:**
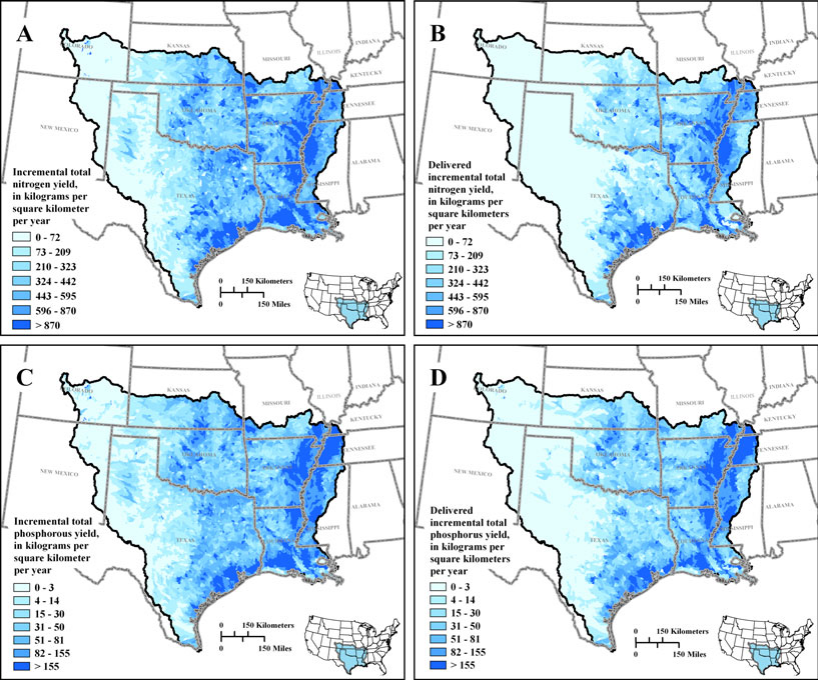
(A) Incremental Total Nitrogen Yield, (B) Delivered Incremental Total Nitrogen Yield, (C) Incremental Total Phosphorus Yield, and (D) Delivered Incremental Total Phosphorus Yield for the Lower Mississippi Texas-Gulf Region. Incremental yields are those generated for individual catchments, and delivered incremental yields are the portion of incremental yields that are delivered to downstream target areas, in this case, to terminal reaches along the Louisiana and Texas coasts.

Incremental and delivered incremental TN and TP yields based on LMTG model output indicated that streams in the eastern part of the LMTG region and streams along the coast delivered more TN and TP than other locations (Figure 3). Reasons for high TN and TP yields in eastern LMTG streams include the following: (1) high rainfall, due to moist and unstable air-mass conditions in the eastern part of the region as opposed to arid conditions in the western and northwestern parts; (2) large inputs from sources such as agricultural fertilizer, livestock manure (confined), and atmospheric deposition (with respect to TN yields only); and (3) higher order streams with larger average flows in eastern than in western streams, and larger average flows lead to less instream loss of nutrients as they are transported to the Gulf of Mexico. Similar to streams in the eastern part of the LMTG region, most streams along the coast are in areas with high rainfall and are commonly larger order streams in which nutrient losses are negligible (with the exceptions being streams along the mid-Texas and lower-Texas coast). Coastal areas include several large population centers, thus nutrient yield of streams in those areas were influenced by inputs from point sources and urban runoff. Also, shorter travel distances in these coastal streams resulted in lower instream loss of nutrients compared to that in streams originating farther inland. Incremental and delivered incremental TN and TP yields for the LMTG region showed dendritic patterns similar to those for the national SPARROW model, indicating that a high percentage of yield is likely to be transported to the northwestern Gulf of Mexico from catchments nearest to large streams or from smaller streams that flow quickly into large streams (Alexander *et al.*, 2008).

On average, model results indicated that more of the P load (about 59%) generated from catchments in the LMTG region was delivered to the Gulf of Mexico than N load (about 48% as shown in Table 2). However, median TN and TP yields from LMTG catchments were 369 and 36 kg/km^2^/year, respectively, indicating that while a higher percentage of P generated in LMTG catchments was delivered to the Gulf, yields from N sources were at least an order of magnitude greater than yields from P sources.

**TABLE 2:** Summary Statistics of Yields and Source Shares From Incremental Catchments From the Lower Mississippi Texas-Gulf Region (2002 conditions)

	Total Nitrogen							Total Phosphorus						
		
			Percentiles							Percentiles				
						
	Mean	SD	10th	25th	50th	75th	90th	Mean	SD	10th	25th	50th	75th	90th
Incremental yield (kg/km^2^/year)	-	-	43	165	369	616	955	-	-	2	10	36	85	185
Source shares (%)														
Atmospheric deposition	42	22	18	25	37	55	76	-	-	-	-	-	-	-
Point sources	4	15	0	0	0	0	9	5	16	0	0	0	0	13
Urban runoff	11	12	2	5	8	13	21	23	20	3	10	18	31	51
Farm fertilizer	20	18	0	4	15	32	48	28	24	0	7	24	45	66
Livestock manure, confined	6	10	0	1	2	6	18	-	-	-	-	-	-	-
Livestock manure, unconfined	17	11	1	7	16	25	32	-	-	-	-	-	-	-
Livestock manure, combination of confined and unconfined	-	-	-	-	-	-	-	23	18	1	8	20	34	48
Phosphorus from sediment from in-channel erosion	-	-	-	-	-	-	-	11	21	0	0	1	13	37
Background sources (forest, wetlands, barren, scrub)	-	-	-	-	-	-	-	9	16	0	1	3	10	23
Percentage of nitrogen/phosphorus load delivered tothe northwestern Gulf of Mexico from all reaches	48	37	0	11	46	88	100	59	35	6	23	63	99	100

Notes: Incremental yields represent the load generated within an incremental watershed (the area that drains directly to a stream reach without passing through another stream reach) divided by the area of the incremental watershed. Mean and standard deviation values for incremental yields are not shown here due to influence of large values in these datasets. The large values are caused by large loads generated in catchments that have very small drainage areas. Source shares represent the contribution from each source as a percentage of the incremental yield.

**FIGURE 4:**
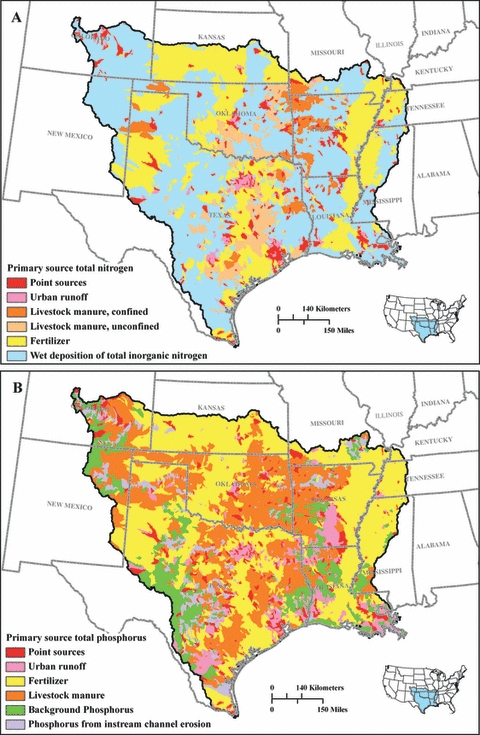
Primary Sources of (A) Total Nitrogen and (B) Total Phosphorus as a Percentage of Incremental Catchment Loads in the Lower Mississippi Texas-Gulf Region.

Model output indicated that the largest source contributions of N to LMTG streams were atmospheric deposition (42%), commercial fertilizer (20%), and livestock manure from unconfined animal feeding operations (17%) (Table 2). The largest source contributions of P to LMTG streams were commercial fertilizer (28%), urban runoff (23%), and livestock manure combined from both confined and unconfined animal feeding operations (23%). Combined urban sources (point sources and urban runoff) contributed about 15% TN and about 28% TP, and P bound to sediment from in-channel erosion contributed about 11% TP to LMTG streams. For TN, model output suggested that the two most dominant sources spatially were atmospheric deposition and fertilizer (Figure 4A). For TP, the two most dominant sources were fertilizer and manure (Figure 4B). Source contributions mentioned here were averages for catchments in the entire LMTG region and could be much higher or lower for any particular catchment.

If all agricultural sources were considered, contributions presented here for the LMTG region (43% for TN and 51% for TP as shown in Table 2) were similar compared to national SPARROW results presented in Smith *et al.* (1997) (48% for TN and 50% for TP) but were lower than those presented in Alexander *et al.* (2008) (58% for TN and 80% for TP). For the agricultural sources, all three models used similar county-level datasets for fertilizer sales and livestock manure contributions (such as those presented in Ruddy *et al.*, 2006) except for different baseline periods. Urban and nonagricultural/nonurban contributions from the LMTG region were not directly comparable to the previous national models. The one major difference between the LMTG models and national models was the contribution of atmospheric deposition N as a percentage of the total N load. Atmospheric deposition N contributed about 42% of the TN load to LMTG streams. Atmospheric deposition contributed only about 18% of the TN load in the two previously presented national models. However, the national models only considered wet deposition of nitrate-N whereas the LMTG-TN models considered wet deposition of TIN which included contributions of nitrate-N and ammonia-N. Smith *et al.* (1997) indicated that total deposition could be 3-4 times wet nitrate deposition. In addition, another study by Alexander *et al.* (2001) indicated that some of the watersheds along the Louisiana and Texas coasts had fairly high export rates for atmospheric N including Calcasieu Lake and Upper Laguna Madre and that atmospheric N was a large percentage of the total N export in Terrebonne Bay and Sabine Lake.

It should be noted that selected source terms included in each model could be incorporating the effects of other parameters correlated with the source terms or could be compensating for source terms that were either not included or were not considered statistically significant (*p* > 0.05). For example, in the TN model, atmospheric deposition was the dominant source. However, there were no background sources of N that were considered in the TN model or were statistically significant (e.g., grass and/or scrubland land-use classifications). The atmospheric deposition term could, therefore, also be representing N from background sources. Another example related to the TN model is that N from crop fixation was not accounted for as a specific source. Datasets that adequately describe N fixation for the LMTG region were unavailable. Fixation as a source of N could be indirectly related to other terms such as atmospheric deposition and spreading of manure. Therefore, it was likely that several of the source coefficients in the TN model indirectly accounted for N from crop fixation. With respect to the TP model, commercially applied fertilizer was the primary source of P for northwestern Mississippi, an area dominated with row crop agriculture (Figure 4B). However, very little P is applied to fields annually in this region due to adequate supply of P in soils (McDowell *et al.*, 1989; Mississippi State University, 2008, 2009). For these areas of the LMTG region, the commercial fertilizer term could be accounting for elevated residual P in soils due to prior P applications and now soils are the source, or the commercial fertilizer term could be accounting for another source of P not included in the TP model. This model limitation could be related to the fact that data available for model input are insufficient to document such things as residual P in soils. Also, fertilizer input datasets were based on county sales data, not actual placement, and were distributed according to agricultural land use in general.

### Loads, Yields, and Sources by Watershed

Model output indicated that the Lower Mississippi River and Atchafalaya River/Terrebonne Bay watersheds delivered the greatest loads to the northwestern Gulf of Mexico, as expected (Table 3). Yields from the LMTG models were compared to those presented in literature, and to accomplish direct comparisons, TN and TP yields were re-computed for four selected watersheds of the MARB and for the entire MARB as follows: Arkansas River, Red River, Lower Mississippi (excluding the Arkansas River), and Atchafalaya River (excluding the Red River) (Table 4). Although the yields were computed for varying time periods and by different methods in these studies, yields generated by the LMTG models were in general agreement with published values. The major difference was the wide variation in yields computed for the Lower Mississippi River basin, which is likely due to how each study computed yields for this basin. In all cases, yields from the Lower Mississippi River basin were the highest of the three selected watersheds and were higher than the yields for the entire MARB. This indicates that, although loads from the Lower Mississippi River basin were a fraction of the MARB load, more load per unit drainage area was being generated from the Lower Mississippi River basin than from other parts of the MARB except for the Upper Mississippi River basin as indicated in Goolsby *et al.* (1999) and Turner and Rabalais (2004). In addition, most of the yield generated from this same area was delivered to the Gulf as previously stated (Figures 3B and 3D).

**TABLE 3:** Watershed load, yield, and source estimates from the Lower Mississippi Texas-Gulf region

Nitrogen model results by watershed										Percent delivered load by source, percent				
	
Watershed^1^	Delivered load, mT/yr	Standard error for delivered load, mT/yr	Lower 90th percentile confidence interval for delivered load, mT/yr	Upper 90th percentile confidence interval for delivered load, mT/yr	Delivered yield, kg/km^2^/yr	Standard error of delivered yield, kg/km^2^/yr	Lower 90th percentile confidence interval for deliverd yield, kg/km^2^/yr	Upper 90th percentile confidence interval for deliverd yield, kg/km^2^/yr	Wet deposition of inorganic nitrogen	Combined industrial and municipal point sources	Nitrogen from urban runoff from developed land use classes	Nitrogen from livestock manure from confined animal feeding operations	Nitrogen from livestock manure from unconfined animal feeding operations	Nitrogen from fertilizer applied to crops
Lake Borgne	3,270	2,320	1,330	8,150	205	145	83	511	41	23	13	4	8	10
Lower Mississippi River	183,000	125,000	54,300	341,000	300^2^	-2	-2	-2	26	19	8	5	10	32
Barataria Bay	1,960	1,360	633	4,380	342	237	110	763	43	20	8	0	8	22
Atchafalaya River/Terrebonne Bay	92,800	63,700	37,500	198,000	355	244	144	758	34	17	8	7	11	22
Mermentau River	3,160	2,260	1,000	6,910	351	252	111	769	34	2	7	0	11	46
Calcasieu River	6,520	4,510	2,170	17,900	591	409	197	1,620	31	34	13	0	8	14
Neches/Sabine Rivers	20,600	14,200	6,750	45,300	381	262	125	835	31	21	15	7	14	11
Trinity River/Galveston Bay	40,500	28,100	13,400	82,100	656	456	217	1,330	10	60	13	1	8	8
Brazos River	24,500	16,800	8,440	56,600	201	138	69	464	15	27	7	3	20	27
Colorado River/Matagorda Bay	16,300	11,200	5,860	28,700	133	92	48	235	17	16	7	3	21	35
San Antonio/Guadalupe Rivers	9,680	6,660	3,310	25,900	361	248	123	966	17	22	10	10	22	19
Aransas River	2,760	1,950	1,080	6,190	431	304	169	965	26	2	9	1	20	42
Nueces River/Corpus Christi Bay	2,330	1,610	788	5,360	52	36	18	120	17	41	14	1	8	20
Upper Laguna Madre	2,230	1,550	764	4,020	145	100	50	261	31	8	9	3	20	30
Lower Laguna Madre	2,440	1,690	869	6,880	228	158	81	642	19	31	14	1	6	30

Phosphorus model results by watershed									Percent delivered load by source, percent					
	
Watershed^1^	Delivered load, mT/yr	Standard error for delivered load, mT/yr	Lower 90th percentile confidence interval for delivered load, mT/yr	Upper 90th percentile confidence interval for delivered load, mT/yr	Delivered yield, kg/km^2^/yr	Standard error of delivered yield, kg/km^2^/yr	Lower 90th percentile confidence interval for deliverd yield, kg/km^2^/yr	Upper 90th percentile confidence interval for deliverd yield, kg/km^2^/yr	Combined industrial and municipal point sources	Phosphorus from urban runoff from developed land use classes	Phosphorus from fertilizer applied to crops	Phosphorus from livestock manure from confined and unconfined feeding operations	Phosphorus attached to sediment from in-channel erosion	Phosphorus from background sources of land use categories
Lake Borgne	701	617	183	1,870	44	39	12	117	22	41	15	11	6	5
Lower Mississippi River	41,200	33,500	6,920	119,000	70^2^	-2	-2	-2	21	16	50	9	3	2
Barataria Bay	401	369	87	949	70	64	15	165	46	16	25	6	5	3
Atchafalaya River/Terrebonne Bay	15,000	12,300	2,820	43,900	58	47	11	168	21	20	36	14	5	4
Mermentau River	923	794	195	2,800	103	88	22	312	2	19	65	10	3	2
Calcasieu River	1,370	1,130	328	3,740	124	102	30	339	26	34	23	9	3	5
Neches/Sabine Rivers	2,960	2,420	618	8,460	55	45	11	156	21	37	15	18	5	5
Trinity River/Galveston Bay	6,230	5,120	1,330	19,000	101	83	22	308	51	29	9	8	2	1
Brazos River	3,280	2,680	666	9,910	27	22	5	81	27	17	32	19	4	1
Colorado River/Matagorda Bay	2,170	1,770	522	5,450	18	15	4	45	11	15	45	22	5	2
San Antonio/Guadalupe Rivers	1,290	1,060	244	3,600	48	39	9	134	25	23	21	22	6	2
Aransas River	404	346	83	1,050	63	54	13	164	2	19	56	17	3	3
Nueces River/Corpus Christi Bay	337	278	64	1,050	8	6	1	24	39	25	19	8	5	3
Upper Laguna Madre	202	166	40	496	13	11	3	32	10	19	45	18	6	2
Lower Laguna Madre	256	211	47	742	24	20	4	69	41	24	28	4	3	1

Notes: LMTG, Lower Mississippi Texas-Gulf region.

1 Selected watersheds were combined as follows: (1) the Lake Borgne watershed includes reaches from the Breton/Chandeleur Sound watershed, (2) the Atchafalaya River/Terrebonne Bay watershed is a combination of the Atchafalaya/Vermillion Bays watershed and the Terrebonne/Timbalier Bays watershed, and (3) the Upper Laguna Madre watershed includes reaches from the Palo Blanco River watershed. In addition, some of the estuarine watershed names do not reflect important rivers or tributaries. For example, the Galveston Bay watershed was revised to be the Trinity River/Galveston Bay watershed. The Lower Mississippi River watershed includes the Arkansas and White Rivers, and the Atchafalaya River/Terrebonne Bay watershed includes the Red River. “Delivered” is defined as loads and yields delivered to the target reaches of the LMTG region which include the terminal reaches of each stream that empties into a bay, estuary, or directly to the Gulf of Mexico along the Louisiana and Texas coasts. For ease of use, “delivered” infers delivered to the Gulf of Mexico.

2 Delivered yield was re-calculated by removing upper Mississippi River loads from LMTG loads, and subsequently, standard error calculations and confidence intervals were not re-calculated.

**TABLE 4:** Estimated yields of total nitrogen and total phosphorus for selected watersheds of the Mississippi-Atchafalaya River Basin.^1^

	Total Nitrogen	Total Phosphorus
		
Watershed	Yield (kg/km^2^/yr)	
Lower Mississippi		
Smith *et al.* (1993)	NA	36
Goolsby *et al.* (1999)	630	58
Turner and Rabalais (2004)	1700	194
LMTG SPARROW models^2^	734	197
Arkansas-White^3^		
Lurry and Dunn (1997)	110	11
Goolsby *et al.* (1999)	130	13
Turner and Rabalais (2004)	141	14
LMTG SPARROW models	111	11
Red^4^		
Goolsby *et al.* (1999)	250	53
Turner and Rabalais (2004)	147	24
LMTG SPARROW models	156	22
Atchafalaya		
Alexander *et al.* (2008)	397	34
LMTG SPARROW models^5^	355	58
Entire Mississippi River^6^		
Smith *et al.* (1993)	NA	38
Lurry and Dunn (1997)	398	34
Goolsby *et al.* (1999)	497	32
Turner and Rabalais (2004)	483	45
Alexander *et al.* (2008)	377	30
LMTG SPARROW models	337	36

Notes: NA, not available; LMTG, Lower Mississippi Texas-Gulf region; SPARROW, SPAtially Referenced Regressions on Watershed attribuites; USGS, U.S. Geological Survey

1 This table is modified from Turner and Rabalais, 2004.

2 Lower Mississippi River yields presented here were computed by subtracting Upper Mississippi and Arkansas River loads and drainage areas from entire Mississippi River loads and drainage areas.

3 USGS gaging station 07263620 Arkansas River at David D Terry Lock and Dam below Little Rock, Arkansas.

4 USGS gaging station 07355500 Red River at Alexandria, Louisiana.

5 Atchafalaya River yields presented here include the Red River drainage area for comparison purposes to Alexander et al. (2008)

6 USGS gaging station 07373420 Mississippi River near St. Francisville, LA.

Although the Lower Mississippi River and Atchafalaya River/Terrebonne Bay watersheds delivered the greatest loads to the northwestern Gulf of Mexico, delivered yields from other watersheds were as high or higher (Table 3) (see also Rebich and Demcheck, 2007). The watersheds with the three highest delivered TN yields were from the Trinity River/Galveston Bay, Calcasieu River, and Aransas River watersheds, while watersheds with the three highest delivered TP yields were from the Calcasieu River, Mermentau River, and Trinity River/Galveston Bay watersheds (Table 3). Yield estimates from the LMTG models for selected Texas and Louisiana coastal watersheds were compared to literature values, namely, Alexander *et al.* (2000) and Dunn (1996) (Table 5). Yield values presented in Alexander *et al.* (2000) were based on national SPARROW results for a baseline year of 1987. Dunn (1996) computed yields for the period 1972-1993 for most of the major rivers that drain from the conterminous U.S. to the Gulf. Because several watersheds were combined for presentation purposes in this paper, yields published by Dunn (1996) were re-computed for comparison purposes for the following watersheds: (1) San Antonio and Guadalupe Rivers, (2) Brays Bayou, Whiteoak Bayou, and Trinity River, and (3) the Neches and Sabine Rivers. Although the yields were computed for varying time periods and by different methods in these studies, yields estimated from the LMTG models compared well to published estimates for the most part. Departures from published values were likely due to how watersheds were delineated for each study and not necessarily a result of land-use changes in a specific watershed that could have caused an increase or decrease in yields. For example, the Galveston Bay watershed was delineated for this study to include not only the Trinity River but also the San Jacinto River [which was excluded in Dunn (1996)], and the Upper Laguna Madre included the Palo Blanco River and Baffin Bay.

**TABLE 5:** Estimated Yields of Total Nitrogen and Total Phosphorus for Selected Watersheds Along the Louisiana and Texas Coasts

	Total Nitrogen	Total Phosphorus
		
Watershed	Yield (kg/km^2^/year)	
Barataria Bay		
Alexander *et al.* (2000)	541	NA
LMTG SPARROW models	342	70
Calcasieu		
Alexander *et al.* (2000)	616	NA
Dunn (1996)	546	34
LMTG SPARROW models	591	124
Neches/Sabine		
Alexander *et al.* (2000)	351	NA
Dunn (1996)	220	15
LMTG SPARROW models	381	55
Trinity/Galveston Bay		
Alexander *et al.* (2000)	468	NA
Dunn (1996)	258	44
LMTG SPARROW models	656	101
Brazos		
Dunn (1996)	130	18
LMTG SPARROW models	201	27
Colorado/Matagorda Bay		
Alexander *et al.* (2000)	123	NA
Dunn (1996)	36	6
LMTG SPARROW models	133	18
San Antonio/Guadalupe		
Dunn (1996)	311	52
LMTG SPARROW models	361	55
Nueces/Corpus Christi Bay		
Alexander *et al.* (2000)	56	NA
Dunn (1996)	31	3
LMTG SPARROW models	52	8
Upper Laguna Madre		
Alexander *et al.* (2000)	717	NA
LMTG SPARROW models	145	13
Lower Laguna Madre		
Alexander *et al.* (2000)	566	NA
LMTG SPARROW models	228	24

Notes: NA, not available; LMTG, Lower Mississippi Texas-Gulf region; SPARROW, SPAtially Referenced Regressions On Watershed attributes.

With respect to TN source contributions within the coastal watersheds, model output indicated that atmospheric deposition was highest (>40%) in the Lake Borgne and Barataria Bay watersheds. The Trinity River/Galveston Bay and Nueces River/Corpus Christi Bay watersheds were highly influenced by urban activities as the combined point source and urban runoff contributions totaled more than 50% of the delivered load. In contrast, the Colorado River/Matagorda Bay and Aransas River watersheds were affected by agricultural activities as the combined fertilizer and livestock manure contributions totaled nearly 60% or more of the delivered load, respectively. With respect to TP source contributions, the Trinity River/Galveston Bay, Nueces River/Corpus Christi Bay, and Lower Laguna Madre watersheds were highly influenced by urban activities as the combined point source and urban runoff contributions totaled more than 60% of the delivered load. In contrast, the Colorado River/Matagorda Bay, Aransas River, and Upper Laguna Madre watersheds were affected by agricultural activities as the combined fertilizer and livestock manure contributions totaled more than 60% of the delivered load. Although statistically significant as sources in the TP model, the combined P contribution from in-channel erosion (sediment) and from background land-use classifications did not total more than 11% of the delivered load for any of the 15 watersheds in the LMTG region.

### Watershed Specific Example

**FIGURE 5:**
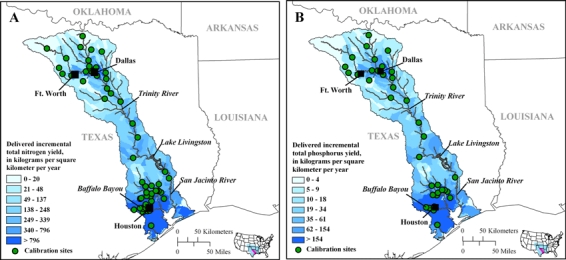
(A) Delivered Incremental Total Nitrogen Yield and (B) Delivered Incremental Total Phosphorus Yield for the Trinity River/Galveston Bay Watershed. Delivered incremental yields are the portion of incremental yields (yields generated for individual catchments) that are delivered to downstream target areas, in this case, to reaches that terminate into Galveston Bay.

Results from the SPARROW models can help resource managers in the LMTG region address critical questions concerning nutrient issues in local watersheds. For example, LMTG model output for the Trinity River/Galveston Bay watershed indicated that pockets of elevated delivered incremental TN and TP yields were located in the upper part of the watershed and near the watershed outlet in close proximity of the cities of Dallas/Ft. Worth and Houston, respectively (Figures 5A and 5B).

Combined urban sources (point sources and urban runoff) were the primary source contributions in this watershed and accounted for about 73% of the TN load and about 80% of the TP load delivered to the outlet of the watershed based on LMTG model output (Table 3). The impact of urban sources has been well documented as to their relative importance to nutrient loadings into Galveston Bay (Land and Shipp, 1996; East *et al.*, 1998; Land *et al.*, 1998; Sneck-Faher *et al.*, 2005; see also Newell *et al.*, 1992; Armstrong and Ward, 1993; Galveston Bay National Estuary Program, 1994), although percentages in other publications were typically lower than those listed here for the LMTG models. For example, point source contributions were 44% TN and 34% TP to Galveston Bay as presented in the Galveston Bay Plan of the Galveston Bay National Estuary Program (1995) while point source contributions based on LMTG model output were 60% TN and 51% TP to Galveston Bay. Higher percentages of point source contributions for the LMTG models were likely artifacts of model calibration indicated by model coefficients for point sources greater than one for both models as previously explained.

The effect of reservoirs on nutrient loads entering Galveston Bay has also been a focus of previous work. Lake Livingston, located on the Trinity River about 175 km above Galveston Bay, was constructed in 1971 for water supply purposes (Phillips and Slattery, 2007) and is considered a significant sink for nutrient loads (van Metre and Reutter, 1995; see also Stanley, 1992; Galveston Bay National Estuary Program, 1994). In their work, van Metre and Reutter (1995) computed reductions in nutrient loads for the period 1974-1991 by subtracting the mean annual nutrient loads from the USGS gaging station 08066500 Trinity River at Romayor located 50 km downstream of the reservoir from mean annual loads computed at USGS gaging station 08065350 Trinity River near Crockett located 110 km upstream of the reservoir. Their work indicated that Lake Livingston reduced TN loads by 35% and TP loads by 65%. Using similar methods, Clingenpeel (2002) indicated a 71% reduction for TN loads and 63% reduction for TP loads for 2000, and a 36% reduction for TN loads and a 58% reduction for TP loads for 2001. Output from the LMTG models indicated a 26% TN load reduction and a 46% TP load reduction over the same portion of the Trinity River that included Lake Livingston as defined in these previous studies. The reduction percentages for the LMTG models were lower than those presented in other studies likely reflecting the fact that LMTG values represent long-term mean annual values. In addition, SPARROW model output includes a direct estimate of reservoir decay computed for each reservoir in the LMTG region, and for Lake Livingston, model output indicated a 37% TN load reduction and a 30% TP load reduction through the Lake. In all cases, Lake Livingston does, in fact, reduce nutrient loads as previously reported, but it does not reduce all nutrient loads generated from upstream locations from entering Galveston Bay.

Much of the focus of nutrient loading to Galveston Bay has centered on the Trinity River due to the fact that it accounts for about 70% of the total Galveston Bay watershed. However, model output indicated an area of elevated TN and TP yield in the western part of the watershed, which includes the San Jacinto River and City of Houston (located on the north shore of Galveston Bay, see Figures 5A and 5B). Although the San Jacinto River flows into the Lake Houston reservoir, which was also built for water supply purposes, the reservoir is located upstream of the City of Houston, and therefore, does not trap nutrients generated within city limits from point sources and urban runoff. The western portion of Galveston Bay has also been characterized as having high nutrient loads which increases eutrophication and phytoplankton community response in this area (East *et al.*, 1998; Quigg and Roehrborn, 2008; Quigg, 2009). Quigg (2009) went on to say that, in 2008, the San Jacinto River watershed was a greater contributor of nutrients to Galveston Bay than was the Trinity River, and that the nutrient inputs from the San Jacinto River reflected inputs from the Houston Ship Channel and urbanization and industrialization from the Houston metropolitan area. Therefore, delivered incremental yields from LMTG model output can help resource managers understand yield contributions for the entire Galveston Bay watershed, not only from the Trinity River, but also the western portions of the watershed.

## Summary and Conclusions

SPARROW models were developed to estimate delivery of TN and TP loads (mass) and yields (mass per unit area of catchment) from streams that drain to the northwestern part of the Gulf of Mexico from the Lower Mississippi, Arkansas-White-Red, and Texas-Gulf (LMTG) region. Load estimates standardized to the 2002 base year from 344 sampled sites for the TN model and from 442 sampled sites for the TP model were used for calibration. Streams in the eastern part of the region and streams along the coast delivered more N and P than other locations in the LMTG region, a consequence of shorter travel times and distances, higher inputs from sources, rainfall patterns, and less instream loss of nutrients (streams in eastern and coastal areas are larger than those in other areas in the LMTG region). The Mississippi River and Atchafalaya River/Terrebonne Bay watersheds, which drain nearly two-thirds of the conterminous U.S. land area, delivered the largest loads of TN and TP to the Gulf of Mexico, as expected, but yields from other watersheds in the LMTG region were as high or higher than those in these two watersheds. The highest delivered TN yields were from the Trinity River/Galveston Bay, Calcasieu River, and Aransas River watersheds, while the highest delivered TP yields were from the Calcasieu River, Mermentau River, and Trinity River/Galveston Bay watersheds.

Simulations made with the LMTG SPARROW models developed here allow for relative watershed-to-watershed comparison of nutrient yields and sources, and results can be scaled to address local watershed issues as well as broader concerns such as the influence of LMTG streams on Gulf hypoxia. Delivered incremental yields and primary sources as identified from LMTG model output, such as those presented for the Trinity River/Galveston Bay watershed, provide a complete picture to assess the origin of elevated nutrient loads and yields and to identify the primary sources of nutrients within those areas. Such information is useful to water resource managers in nutrient criteria and total maximum daily load development, as well as nutrient reduction strategies to protect downstream sea grass beds and other aquatic resources in bays and estuaries along the Louisiana and Texas coasts.

It should be noted that uncertainty increases in load estimates and source allocations, especially at very small scales or for very small tributaries, due to limitations in the datasets used to complete model calibration and predictions. The LMTG SPARROW models could be improved by using a more refined digital stream reach network, which would allow for better source and land-to-water delineations, use of more monitoring stations for calibration, and better estimates of nutrient transport and decay. LMTG models also could be improved by using updated datasets such as confirmation of precise tributary locations and load estimates for municipal and industrial point sources.

## SUPPORTING INFORMATION

**Data S1.** Development of the LMTG SPARROW models, maps of final sources and land-to-water delivery variables used, and maps of delivered incremental yield for watersheds in the LMTG region not presented in the article are available as part of the online paper.

Please note: Neither AWRA nor Wiley-Blackwell is responsible for the content or functionality of any supporting materials supplied by the authors. Any queries (other than missing material) should be directed to the corresponding author for the article.

## References

[b1] Alexander RB, Smith RA, Schwarz GE (2000). Effect of Stream Channel Size on the Delivery of Nitrogen to the Gulf of Mexico. Nature.

[b2] Alexander RB, Smith RA, Schwarz GE, Boyer EW, Nolan JV, Brakebill JW (2008). Differences in Phosphorus and Nitrogen Delivery to the Gulf of Mexico From the Mississippi River Basin. Environmental Science and Technology.

[b3] Alexander RB, Smith RA, Schwarz GE, Preston SD, Brakebill JW, Srinivasan R, Pacheco PA (2001). Atmospheric Nitrogen Flux From the Watersheds of Major Estuaries of the United States – An Application of the SPARROW Watershed Model. *In*: Nitrogen Loading in Coast Water Bodies – An Atmospheric Perspective Coastal and Estuarine Studies. American Geophysical Union.

[b4] Armstrong NE, Ward GH (1993). Point Source Loading Characterization of Galveston Bay.

[b5] Aulenbach BT, Buxton HT, Battaglin WA, Coupe RH (2007). Streamflow and Nutrient Fluxes of the Mississippi-Atchafalaya River Basin and Subbasins for the Period of Record Through 2005. http://toxics.usgs.gov/pubs/of-2007-1080/index.html.

[b6] Baumgardner RE, Lavery TF, Rogers CM, Isil SS (2002). Estimates of the Atmospheric Deposition of Sulfur and Nitrogen Species: Clean Air Status and Trends Network, 1990-2000. Environmental Science and Technology.

[b7] Beaulac MN, Reckhow KH (1982). An Examination of Land Use – Nutrient Export Relationships. Water Resources Bulletin.

[b8] Beven KJ, Kirkby MJ (1979). A Physically Based, Variable Contributing Area Model of Basin Hydrology. Hydrological Sciences Bulletin.

[b9] Brakebill JW, Wolock DM, Terziotti SE Digital Hydrologic Networks Supporting Applications Related to Spatially Referenced Regression Modeling. Journal of the American Water Resources Association.

[b10] Bricker S, Longstaff B, Dennison W, Jones A, Boicourt K, Wicks C, Woerner J (2007). Effects of Nutrient Enrichment in the Nation's Estuaries – A Decade of Change.

[b11] Burkart MR, James DE (1999). Agricultural-Nitrogen Contributions to Hypoxia in the Gulf of Mexico. Journal of Environmental Quality.

[b12] Clement C, Bricker SB, Pirhalla DE, Pam Rubin (2001). Eutrophic Conditions in Estuarine Waters. NOAA's State of the Coast Report.

[b13] Clingenpeel GC, Jenson P, Lee K, Su Y, Garrett T, Ward G, Clingenpeel G (2002). Trinity Basin Nutrient Loads. http://www.tceq.texas.gov/assets/public/permitting/watersupply/water_rights/eflows/01052009sac_handout_pj2.pdf.

[b14] Coupe RH (2002). Nitrogen and Phosphorus Concentrations and Fluxes of Stream in the Mississippi Embayment Study Unit, 1996-98.

[b15] Davis JV, Bell RW (1998). Water-Quality Assessment of the Ozark Plateaus Study Unit, Arkansas, Kansas, Missouri, and Oklahoma—Nutrients, Bacteria, Organic Carbon, and Suspended Sediment in Surface Water, 1993-95.

[b16] Demcheck DK, Tollett RW, Mize SV, Skrobialowski SC, Fendick RB, Swarzenski CM, Porter S (2004). Water Quality in the Acadian-Pontchartrain Drainages, Louisiana and Mississippi, 1999-2001.

[b17] Dortch Q, Peterson TD, Achee S, Furr KL, Turner RE, Justic D, Dortch Q, Rabalais NN (2001). Phytoplankton, Cyanobacterial Blooms, and N2 Fixation in Years With and Without Mississippi River Diversions. Nitrogen Loading Into Lake Pontchartrain.

[b18] Dortch Q, Turner RE, Parsons ML, Rabalais NN, Rozas LP, Nyman JA, Profit CE, Rabalais NN, Reed DJ, Turner RE (1999). What Is the Threat of Harmful Algal Blooms in Louisiana Coastal Waters?. Recent Research in Coastal Louisiana-Natural System Function and Response to Humans Influences.

[b19] Dunn DD (1996). Trends in Nutrient Inflows to the Gulf of Mexico From Streams Draining the Conterminous United States, 1972-93.

[b20] East JW, Paul EM, Porter SD (1998). Nutrient Loading and Selected Water-Quality and Biological Characteristics of Dickinson Bayou Near Houston, Texas, 1995-1997.

[b21] Elliott EM, Kendall C, Wankel SD, Burns DA, Boyer EW, Harlin K, Bain DJ, Butler TJ (2007). Nitrogen Isotopes as Indicators of NOx Sources Contributions to Atmospheric Deposition Across the Midwestern and Northeastern United States. Environmental Science and Technology.

[b22] Galveston Bay National Estuary Program (1994). The State of the Bay – A Characterization of the Galveston Bay Ecosystem.

[b23] Galveston Bay National Estuary Program (1995). The Galveston Bay Plan.

[b24] García AM, Hoos AB, Terziotti S A Regional Modeling Framework of Phosphorus Sources and Transport in Streams of the Southeastern United States. Journal of the American Water Resources Association.

[b25] Ging PB (1999). Water-Quality Assessment of South-Central Texas – Descriptions and Comparisons of Nutrients, Pesticides, and Volatile Organic Compounds at Three Intensive Fixed Sites, 1996-98.

[b26] Goolsby DA, Battaglin WA (2000). Nitrogen in the Mississippi River Basin – Estimating Sources and Predicting Flux to the Gulf of Mexico.

[b27] Goolsby DA, Battaglin WA, Lawrence GB, Artz RS, Aulenbach BT, Hooper RP, Kenney DR, Stensland GJ (1999). Flux and Sources of Nutrients in the Mississippi-Atchafalaya River Basin.

[b28] Haggard BE, Masoner JR, Becker CJ (2003). Percentile Distributions of Median Nitrite Plus Nitrate as Nitrogen, Total Nitrogen, and Total Phosphorus Concentrations in Oklahoma Streams, 1973-2001.

[b29] Hamilton PA, Myers DN, Erwin ML (2005). National Water-Quality Assessment Program, Cycle II – Regional Assessments of Aquifers and Streams and Rivers.

[b30] Holland EA, Braswell BH, Sulzman J, Lamarque JF (2005). Nitrogen Deposition on the United States and Western Europe: Synthesis of Observations and Models. Ecological Applications.

[b31] Hoos AB, McMahon G (2009). Spatial Analysis of In-Stream Nitrogen Loads and Factors Controlling Nitrogen Delivery to Streams in the Southeastern United States Using Spatially Referenced Regression on Watershed Attributes (SPARROW) and Regional Classification Frameworks. Hydrological Processes by Wiley InterScience.

[b32] Land LF, Moring JB, van Metre PC, Reutter DC, Mahler BJ, Shipp AA, Ulery RL (1998). Water-Quality in the Trinity River Basin, 1992-1995.

[b33] Land LF, Shipp AA (1996). Water-Quality Assessment of the Trinity River Basin, Texas – Nutrients in Streams Draining an Agricultural and an Urban Area, 1993-95.

[b34] Louisiana Universities Marine Consortium (2010). Hypoxia in the Northern Gulf of Mexico, Research: Shelfwide Cruises. http://www.gulfhypoxia.net/Research/Shelfwide%20Cruises/.

[b35] Lurry DL, Dunn DD (1997). Trends in Nutrient Concentration and Load for Streams in the Mississippi River Basin, 1974-94.

[b36] Maupin MA, Ivahnenko T Nutrient Loadings to Streams of the Continental United States From Municipal and Industrial Effluent. Journal of the American Water Resources Association.

[b37] McDowell LL, Willis GH, Murphree CE (1989). Nitrogen and Phosphorus Yields in Run-off From Silty Soils in the Mississippi Delta, USA. Agriculture, Ecosystems and Environment.

[b38] van Metre PC, Reutter DC (1995). Water-Quality Assessment of the Trinity River Basin, Texas – Analysis of Available Information on Nutrients and Suspended Sediments, 1974-91.

[b39] Mississippi River/Gulf of Mexico Watershed Nutrient Task Force (2008). Gulf Hypoxia Action Plan 2008 for Reducing, Mitigating, and Controlling Hypoxia in the Northern Gulf of Mexico and Improving Water Quality in the Mississippi River Basin.

[b40] Mississippi State University (2008). Phosphorus in Mississippi Soils: Mississippi State University Extension Service, MSUCares, Information Sheet 871. http://msucares.com/pubs/infosheets/is0871.pdf.

[b41] Mississippi State University (2009). Soybeans-Liming and Fertilization: Mississippi State University Extension Service, MSUCares, Information Sheet 873. http://msucares.com/pubs/infosheets/is0873.htm.

[b42] Mize SV, Demcheck DK (2009). Water Quality and Phytoplankton Communities in Lake Pontchartrain During and After the Bonnet Carre Spillway Opening, April to October 2008. Geo-Marine Letters.

[b43] Newell CJ, Rifai HS, Bedient PB (1992). Characterization of Non-Point Sources and Loadings to Galveston Bay.

[b44] NOAA (National Oceanic and Atmospheric Administration) (2007). NOAA's Coastal Geospatial Data Project. http://coastalgeospatial.noaa.gov/.

[b45] NOAA (National Oceanic and Atmospheric Administration) (2010). NOAA's State of the Coast – Ecosystems – Nutrient Pollution and Hypoxia – Everything Is Upstream of the Coast. http://stateofthecoast.noaa.gov/hypoxia/welcome.html.

[b46] Owenby J, Heim R, Burgin M, Ezell D (2001). Climatography of the U.S., no. 81 – Supplement 3 – Maps of Annual 1961-1990 Normal Temperature, Precipitation and Degree Days. http://www.ncdc.noaa.gov/oa/documentlibrary/clim81supp3/clim81.html.

[b47] Owens PN, Walling DE (2002). The Phosphorus Content of Fluvial Sediment in Rural and Industrialized River Basins. Water Research.

[b48] van der Perk M, Owens PN, Deeks LK, Rawlins BG (2006). Streambed Sediment Geochemical Controls on Instream Phosphorus Concentrations During Baseflow. Water, Air, and Soil Pollution.

[b49] Phillips JD, Slattery MC (2007). Downstream Trends in Discharge, Slope, and Stream Power in a Lower Coastal Plain River. Journal of Hydrology.

[b50] Preston SD, Alexander RB, Schwarz GE, Crawford CG Factors Affecting Stream Nutrient Loads: A Synthesis of Regional SPARROW Model Results for the Continental United States. Journal of the American Water Resources Association.

[b51] Preston SD, Alexander RB, Woodside MD, Hamilton PA (2009). SPARROW Modeling – Enhancing Understanding of the Nation's Water-Quality.

[b52] Preston SD, Brakebill JW (1999). Application of Spatially Referenced Regression Modeling for the Evaluation of Total Nitrogen Loading in the Chesapeake Bay Watershed.

[b53] Quigg AS (2009). Phytoplankton Responses to Freshwater Inflows in the Trinity-San Jacinto Estuary.

[b54] Quigg AS, Roehrborn L (2008). Spatial and Temporal Distributions of Planktonic Diatoms in a Subtropical Bayou. Texas Journal of Science.

[b55] Rabalais NN, Wiseman WJ, Turner RE, SenGupta BK, Dortch Q (1996). Nutrient Changes in the Mississippi River and Responses on the Adjacent Continental Shelf. Estuaries.

[b56] Rebich RA, Demcheck DK (2007). Trends in Nutrient and Sediment Concentrations and Loads in Major River Basins of the South-Central United States, 1993-2004.

[b57] Robertson DM, Schwarz GE, Saad DA, Alexander RB (2009). Incorporating Uncertainty Into the Ranking of Sparrow Model Nutrient Yields From Mississippi/Atchafalaya River Basin Watersheds. Journal of the American Water Resources Association.

[b58] Ruddy BC, Lorenz DL, Mueller DK (2006). County-Level Estimates of Nutrient Inputs to the Land Surface of the Conterminous United States, 1982-2001.

[b59] Saad DA, Schwarz GE, Robertson DM, Booth NL A Multi-Agency Nutrient Dataset Used to Estimate Loads, Improve Monitoring Design, and Calibrate Regional Nutrient SPARROW Models. Journal of the American Water Resources Association.

[b60] Schwarz GE (2008). A Preliminary SPARROW Model of Suspended Sediment for the Conterminous United States. http://pubs.usgs.gov/of/2008/1205.

[b61] Schwarz GE, Hoos AB, Alexander RB, Smith RA (2006). The SPARROW Surface Water-Quality Model – Theory, Application, and User Documentation.

[b62] Seaber PR, Kapinos FP, Knapp GL (1987). Hydrologic Unit Maps.

[b63] Smith RA, Alexander RB (2000). Sources of Nutrients in the Nation's Watersheds. Managing Nutrients and Pathogens From Animal Agriculture, Proceedings From the Natural Resource, Agriculture, and Engineering Service Conference for Nutrient Management Consultants, Extension Educators, and Producer Advisors, March 28-30, 2000.

[b64] Smith RA, Alexander RB, Lanfear KJ, Paulson RW, Chase EB, Williams JS, Moody DW (1993). Stream Water Quality in the Conterminous United States – Status and Trends of Selected Indicators During the 1980s.

[b65] Smith RA, Schwarz GE, Alexander RB (1997). Regional Interpretation of Water-Quality Monitoring Data. Water Resources Research.

[b66] Sneck-Faher DA, Milburn MS, East JW, Oden JH (2005). Water-Quality Assessment of Lake Houston Near Houston, Texas, 2000-2004.

[b67] Stanley DW (1992). Historical Trends: Water Quality and Fisheries: Galveston Bay.

[b68] Thronson A, Quigg A (2008). Fifty-Five Years of Fish Kills in Coastal Texas. Estuaries and Coasts.

[b69] Tortorelli RL (2008). Nutrient Concentrations, Loads, and Yields in the Eucha-Spavinaw Basin, Arkansas and Oklahoma, 2002-2006.

[b70] Turner RE, Rabalais NN (2004). Suspended Sediment, C, N, P, and Si Yields From the Mississippi River Basin. Hydrobiologia.

[b71] U.S. Census Bureau (2001). Census 2000 PHC-T-3, Ranking Tables for Metropolitan Areas, 1990 and 2000, Table 3, Metropolitan Areas Ranked by Population, 2000. http://www.census.gov/population/www/cen2000/briefs/phc-t3/tables/tab03.pdf.

[b72] USDA (U.S. Department of Agriculture) (1994). State Soil Geographic (STATSGO) Data Base, Data Use Information.

[b73] USDA-NRCS (U.S. Department of Agriculture, Natural Resources Conservation Service) (2010). Mississippi River Basin Healthy Watersheds Initiative. http://www.nrcs.usda.gov/programs/mrbi/mrbi_overview.html.

[b74] USEPA (U.S. Environmental Protection Agency) (1996). USEPA Reach File Version 1.0 (RF1) for the Conterminous United States (CONUS). http://www.epa.gov/waters/doc/rf1_meta.html#Citation%20Information.

[b75] USEPA (U.S. Environmental Protection Agency) (2009). Water Discharge Permits (PCS). http://www.epa.gov/enviro/html/pcs/index.html.

[b76] USEPA (U.S. Environmental Protection Agency) (2010). Mississippi River Gulf of Mexico Watershed Nutrient Task Force: Hypoxia 101. http://www.epa.gov/owow_keep/msbasin/hypoxia101.htm.

[b77] USGS (U.S. Geological Survey) (2010). SPARROW Frequently Asked Questions. http://water.usgs.gov/nawqa/sparrow/FAQs/faq.html.

[b78] Wieczorek ME, Lamotte AE (2011). Attributes for MRB_E2RF1 Catchments by Major River Basins in the Conterminous United States. U.S. Geological Survey Digital Data Series DS-491. http://water.usgs.gov/nawqa/modeling/rf1attributes.html.

