# Supplementary material for: Sources and Delivery of Nutrients to the Northwestern Gulf of Mexico from Streams in the South-Central United States
Source: J Am Water Resour Assoc. 2011 Oct;47(5):1061–86. doi: 10.1111/j.1752-1688.2011.00583.x (PMC3307634; doi:10.1111/j.1752-1688.2011.00583.x)
Supplement: Supplementary file 1 [file jawr0047-1061-SD1.pdf]

SUPPLEMENTAL INFORMATION FOR THE ARTICLE:

SOURCES AND DELIVERY OF NUTRIENTS TO THE NORTHWESTERN GULF OF MEXICO FROM STREAMS IN THE SOUTH-CENTRAL UNITED STATES

Richard Rebich[1], Natalie Houston[2], Scott Mize[3], Daniel Pearson, Patty Ging[2], and Evan Hornig[2]

[1] Corresponding author, U.S. Geological Survey, Jackson, MS, *rarebich@usgs.gov*
[2] U.S. Geological Survey, Austin, TX
[3] U.S. Geological Survey, Baton Rouge, LA

In the following sections, we present supporting information on: (1) methods used in developing the total nitrogen (TN) and total phosphorus (TP) SPARROW models; (2) maps of final sources and land-to-water delivery variables used; and (3) maps of delivered incremental yield for watersheds in the Lower Mississippi, Arkansas-White-Red, and Texas-Gulf (LMTG) region not presented in the article.

DETAILED METHODS DESCRIPTIONS USED IN SPARROW MODELING

This section includes: a brief description of the SPARROW model: the reach network used for the LMTG models; the site screening and collocation process; and computations used for delivered load and yield estimates for LMTG watersheds.

SPARROW Model Details

SPARROW is a spatially explicit watershed model that uses a hybrid statistical/mechanistic approach to estimate nutrient sources, transport, and transformation in terrestrial and aquatic ecosystems of watersheds under long-term steady-state conditions (Smith *et al.*, 1997; Alexander *et al.*, 2008). SPARROW includes non-conservative transport, mass-balance constraints, and water flowpaths defined by topography, streams, and reservoirs, based on a stream-reach network with delineated reach catchments. The model-estimated flux leaving each reach (i) in SPARROW, F_i^*, is given by

$$F_i^* = \left[\left(\sum_{j \in J(i)} F_j{}' \right) A(Z_i{}^S, Z_i{}^R; \theta_S, \theta_R) + \left(\sum_{n-1}^{Ns} S_{n,i} \alpha_n D_n (Z_i{}^D; \theta_D) \right) A'(Z_i{}^S Z_i{}^R; \theta_S, \theta_R) \right] \varepsilon_i \quad (1)$$

The first summation term in the above equation represents the total load delivered to reach *i* from upstream reaches, where F_j' is the measured load if the upstream reach is monitored or the model-estimated load if it is not. The A(·) term represents any stream delivery factors that cause load to be lost as it travels along the reach. Within this term, the Z^S and Z^R vectors represent losses in measured stream and reservoirs, respectively (with corresponding coefficient vectors, Θ_s and Θ_r). The second summation term represents the amount of the within-reach load introduced to stream reach *i*. This term is composed of load originating in individual modeled sources, each source being indexed by $n=1,\ldots N_S$. Each source has a source variable, S_n , and a source coefficient, α_n, that measures the intensity of source contribution. The function $D_n(\cdot)$ represents land-to-water delivery factors, and, coupled with the coefficient, represents the rate at which the source variable is converted to nutrient mass delivered to streams. The last term in the equation, the function A'(·), represents the fraction of the load originating in and delivered to reach *i* that is transported to the reach's downstream node. If reach *i* is a stream reach (as opposed to a reservoir reach), the N load or P load introduced to reach *i* from the incremental drainage for reach *i* is attenuated to receive the square root of the reach's full in-stream delivery. For reservoir reaches, the assumption is made that the nutrient mass receives the full attenuation, which is tabulated as a reach attribute. The multiplicative error term in equation (1), ε_i , is applicable in cases where reach *i* is a monitored reach; the error is assumed to be independent and identically distributed across independent catchments in the intervening drainage between stream monitoring sites.

Statistical terms computed during each SPARROW model run that describe model performance and fit include the sum of squared errors (SSE), the mean square

error (MSE), the root mean square error (RMSE), and three coefficients of determination, R^2, adjusted R^2, and yield R^2 (all based on log units, Schwarz *et al.*, 2006). The SSE is the squared value of the estimated residual (actual minus predicted), times its weight, and summed over all monitored reaches. The MSE is the SSE divided by the number of degrees of freedom for the error, or DF Error. The DF error statistic is the difference between the number of observations (sampled locations) and the number of degrees of freedom in the model (number of terms used). The RMSE is the square root of the MSE. The R^2 is the standard coefficient of determination related to the model residual, the measured flux for a particular observation, and the average flux over all observations (described by Judge *et al.*, 1985). R^2 is the portion of the variance in the load data accounted by the independent variables in each model. The adjusted R^2 applies a degree of freedom adjustment to the R^2 statistic. The R^2 and adjusted R^2 terms tend to be large due to the fact that much of the variation in the dependent variable is associated with the size of the drainage area upstream of the monitored reach and that drainage area is highly correlated with source variables; therefore, a high R^2 does not necessarily mean good model fit for smaller basins. Goodness of fit is better described by yield R^2, which is the logarithm of contaminant yield. Yield R^2 is based on applying a drainage area adjustment to the R^2 to account for scaling effects due to drainage area; therefore, yield R^2 is typically lower than R^2 (Schwarz *et al.*, 2006).

Reach Network Details

There were 8,375 stream reaches in the reach network used for the TN and TP SPARROW models of the LMTG region. The reach network was based on the

1:500,000-scale enhanced River Reach File 2.0 (eRF1_2) reach network for the conterminous United States (U.S.) (Brakebill *et al.*, this issue). The eRF1_2 reach network included attributes that describe basin characteristics, channel morphology, and hydraulic properties for each stream reach in the LMTG region—such as estimates of mean streamflow, mean velocity, reach length, and travel time—and reservoirs—such as surface area and outflow. The mean streamflow attributes in the eRF1_2 reach network were revised using an alternate mean streamflow based on an interpolation of USGS streamgage estimates from 1975 to 2007, with extrapolation of streamflow upstream of gages based on runoff estimated at downstream or neighboring stations and apportioned to the land surface according to the eRF1_2 catchments (David Wolock, U.S. Geological Survey, written commun., 2008; Brakebill *et al.*, this issue). Catchments were delineated for each reach from 1-km digital elevation models (DEMs; Brakebill *et al.*, this issue).

Site Screening and Collocation Process

N and P concentration data were retrieved from the U.S. Geological Survey National Water Information System (NWIS) and from the U.S. Environmental Protection Agency Storage and Retrieval System (USEPA-STORET, both Legacy and Modernized, USEPA, 2004). All data from the USGS-NWIS system were considered to be usable for the LMTG SPARROW models because of defined quality assurance protocols (USGS, variously dated). One of the primary uses of the STORET database is for States to input ambient data collected in their State that are similarly collected using State-defined quality assurance protocols. The STORET database was upgraded from Legacy to Modernized during fiscal year 2000. After careful inspection of the combined STORET

data for the LMTG region, it became apparent that some States either did not populate the Legacy database prior to 2000 or did not continue to populate the Modernized STORET after 2000. So, State database retrievals were requested for the States of Mississippi, Louisiana, Texas, Tennessee, Oklahoma, and Kansas (note: data from the State of Arkansas were previously incorporated into the USGS database). Data quality from State databases were assumed to be sufficient for combining with NWIS and USEPA-STORET data although sampling and quality assurance protocols were likely different. Readers are referred to USGS-NWIS, USEPA-STORET, and individual States to obtain concentration data used at specific sites.

Direct measurement of TN and TP concentrations were used for load estimation as well as combinations or substitutions of component N and P species if a direct measurement was not available; for example, if a direct measure of TN was not available, then the sum of ammonia plus organic N (Kjeldahl nitrogen) and nitrite plus nitrate was substituted for TN. Computer programs were written in SAS programming language (SAS, 2003) to compute TN concentrations from other N species when necessary and to eliminate redundant data from the various databases once combined (Saad *et al.,* this issue).

Sites were deleted if located in bays, estuaries, reservoirs, lakes, or inter-coastal canals, or if they could not be located on the eRF1_2 reach network. There were many cases where different agencies collected data at the same location or in close proximity to each other. Sites were considered collocated if they were within 1,000 meters of each other. There were 43 sites that were also considered collocated that were more than 1,000 meters apart but had no major tributaries entering between them (drainage area

ratios were between 0.9 and 1.1). Concentration data were combined for all collocated sites. After the Federal and State databases were combined and the collocation process was completed, there were 22,012 sites where there was at least one TN or TP concentration.

Sites selected for model calibration were screened using criteria such as, but not limited to: each site must have a minimum of 25 samples; each site must have a minimum of 3 samples per season (winter, spring, summer, and fall); and the sampling period at each site must terminate within a specific number of years of the base year of 2002. For calibration sites that had greater than 5 years of data, the sampling period must terminate within 7 years of 2002; for calibration sites that had greater than two years but less than 5 years of data, the sampling period must terminate within 2 years of 2002. There were 1,879 sites that met these criteria for either TN or TP. The screened sites were then plotted along with National Hydrography Dataset (Brakebill *et al.,* this issue) flow gages, and then a Geographic Information System computer program was used to match the screened sites with a flow gage. Specifically, the sampling station must be on the same stream, and the drainage area ratio of the sampling station to the flow station must be within 0.5 to 2. Using these match criteria, 690 screened sites were matched with a flow gage.

All flow data were from USGS flow gages with the exception of two sites from the U.S. Army Corps of Engineers: flow data for the Mississippi River at Tarbert Landing was used for load calculations for USGS site 07373420 Mississippi River near St. Francisville, LA, and flow data for the Atchafalaya River near Simmesport, LA, was used for load calculations for USGS site 07381496 Atchafalaya River at Melville, LA. Figure

S1 shows the ranges of drainage area and estimated loads for sites used in the TN and TP models. The drainage areas for the sites in both models ranged from about 23 to nearly 3 million square kilometers (Mississippi River), and the median drainage area for sites in both models was about 1,800 square kilometers indicating that the model calibration sites were representative of small headwater streams to very large rivers.

Loads of TN, TP, or both were estimated for the 690 matched sites using Fluxmaster software (Schwarz *et al.*, 2006). Further manual screening criteria included, but were not limited to, deleting sites if the following occurred:

- Large temporal gaps (greater than 5 years) in the concentration data set;
- Concentration data were more than 70% censored; and
- Flow data were not inclusive of the target year 2002.

Once these final data screenings were completed, there were a total of 468 unique sites, of which there were 344 for TN and 442 for TP load estimation (Table S1). Of the 468 unique sites, 318 provided both TN and TP load estimates, 26 provided only TN load estimates, and 124 provided only TP load estimates. Of the 468 unique sites, 264 sites were unique to the USGS, 162 sites were unique to individual States, and 42 were sites where both the USGS and State agency data were combined.

Computations Used for Accumulated Delivered Load and Yield Estimates for LMTG Watersheds

A custom SAS program was written to provide accumulated delivered loads and yield estimates for each watershed, and standard errors and confidence limits for these load and yield estimates (Greg Schwarz, U.S. Geological Survey, written communication, July 2009). As part of this process, the analysis first delivers catchment loads to a

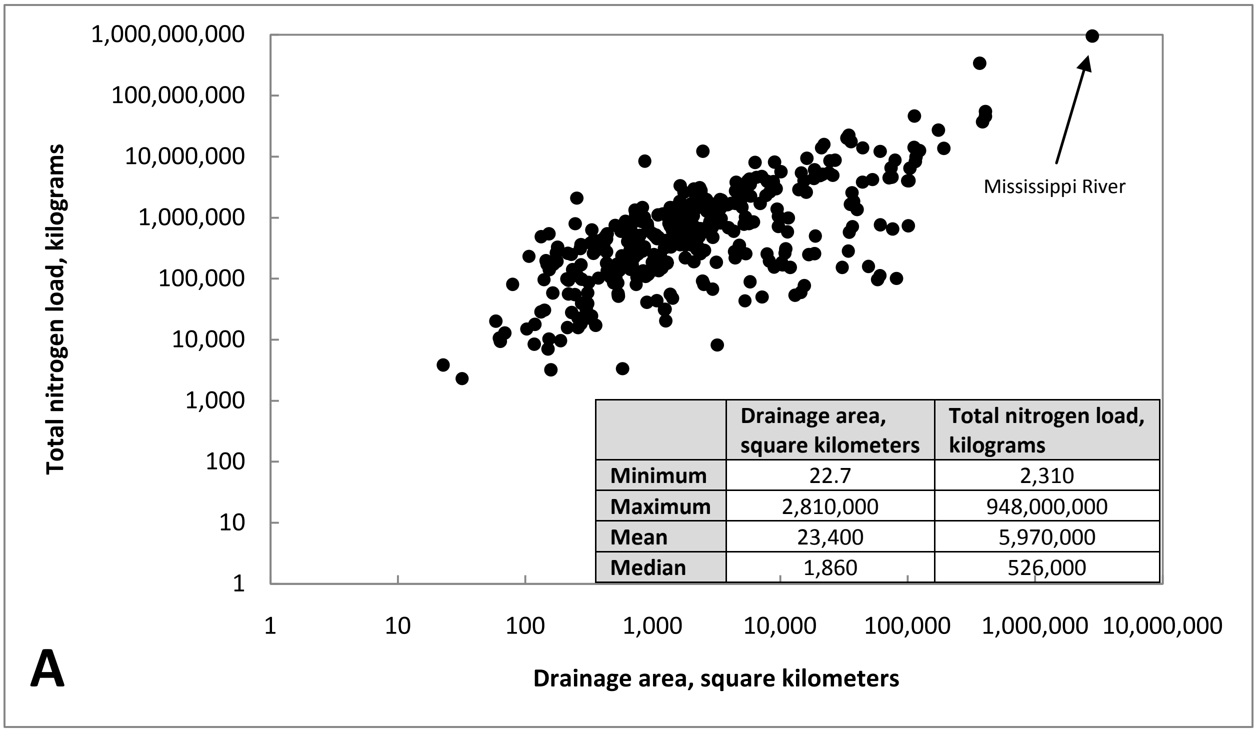

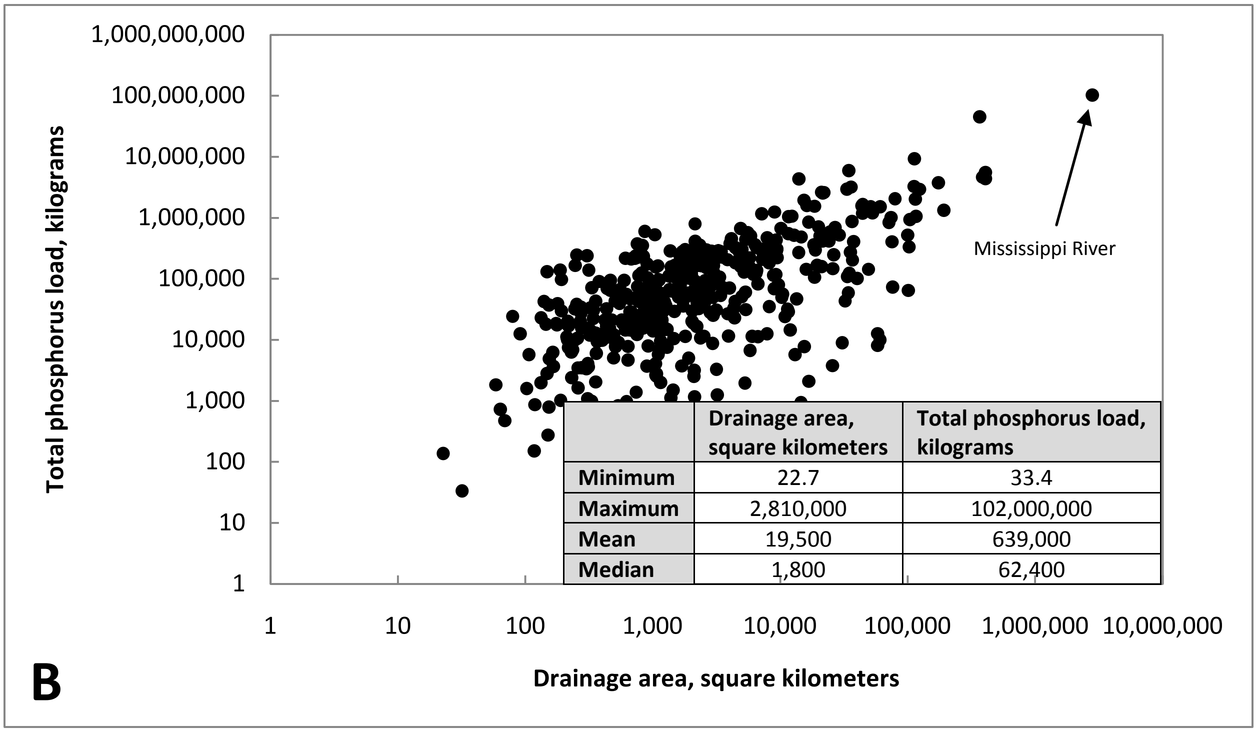

Figure S1 – Drainage area plotted with load estimates for calibration sites used in the (a) total nitrogen and (b) total phosphorus Lower Mississippi Texas-Gulf SPARROW models. Load estimates are the long-term mean annual load standardized to the 2002 base year, which means that the estimate of the mean nutrient load is one that would have occurred in 2002 if mean annual flow conditions from a much longer period of time had prevailed (in this case, 1980 to 2002).

Table S1. Sites used for load estimation for Lower Mississippi Texas-Gulf SPARROW models.

Federal or State agency	Number of sites where loads were estimated
U.S. Geological Survey	264
State of Arkansas*	6
State of Colorado	3
State of Kansas	25
State of Louisiana	22
State of Mississippi	1
State of Oklahoma	27
State of Tennessee	2
State of Texas	76
Sites where U.S. Geological Survey and State data were combined	42
Sites where both total nitrogen and total phosphorus loads were estimated	318
Sites where only total nitrogen loads were estimated	26
Sites where only total phosphorus loads were estimated	124
Total number of sites where total nitrogen loads were estimated	344
Total number of sites where total phosphorus loads were estimated	442
Total number of sites	468

*Note: These State of Arkansas data were from the U.S. Environmental Protection Agency's Storage and Retrieval System (STORET). Other data from the State of Arkansas were incorporated into the U.S. Geological Survey database and are not distinguished in this table.

common downstream target (assuming all reaches in the watershed have the same downstream target). The delivered catchment loads are then accumulated and, if a delivered estimate is not requested, the delivery factor for the most downstream reach in the aggregation unit, as determined by the reach with the largest drainage area, is used to "un-deliver" the accumulated load. In this way, if delivered load is not requested, the estimated load represents the load leaving the aggregation area. Complications may arise if there are multiple outlets for the watershed that have different values of the delivery fraction variable. In these cases, the aggregation effectively accumulates the load leaving each outlet and "delivers" it to the outlet corresponding to the reach with the largest upstream drainage area.

MAPS OF FINAL SOURCE AND LAND-TO-WATER DELIVER VARIABLES

Maps of final source and land-to-water delivery terms used in the LMTG TN and TP SPARROW models are based on aggregated datasets (Wieczorek and Lamotte, 2011; unless otherwise noted all spatial data in this article are from this source).. Figure S2 is a plot of land use in the LMTG region. The largest land use grouping is pasture and grassland at 32% followed by forested (19%), cropped (19%), barren and scrub (16%), wetlands (7%), developed residential (5%) and open water (2%). Developed residential land use was used as a surrogate for urban runoff in both models; forested, barren and scrub, and wetlands land use categories were combined and used as a surrogate for background P in the TP model.

Figures S3 and S4 are plots of point source and fertilizer loadings used in both models. Figures S5 and S6 are plots of livestock manure from confined and unconfined

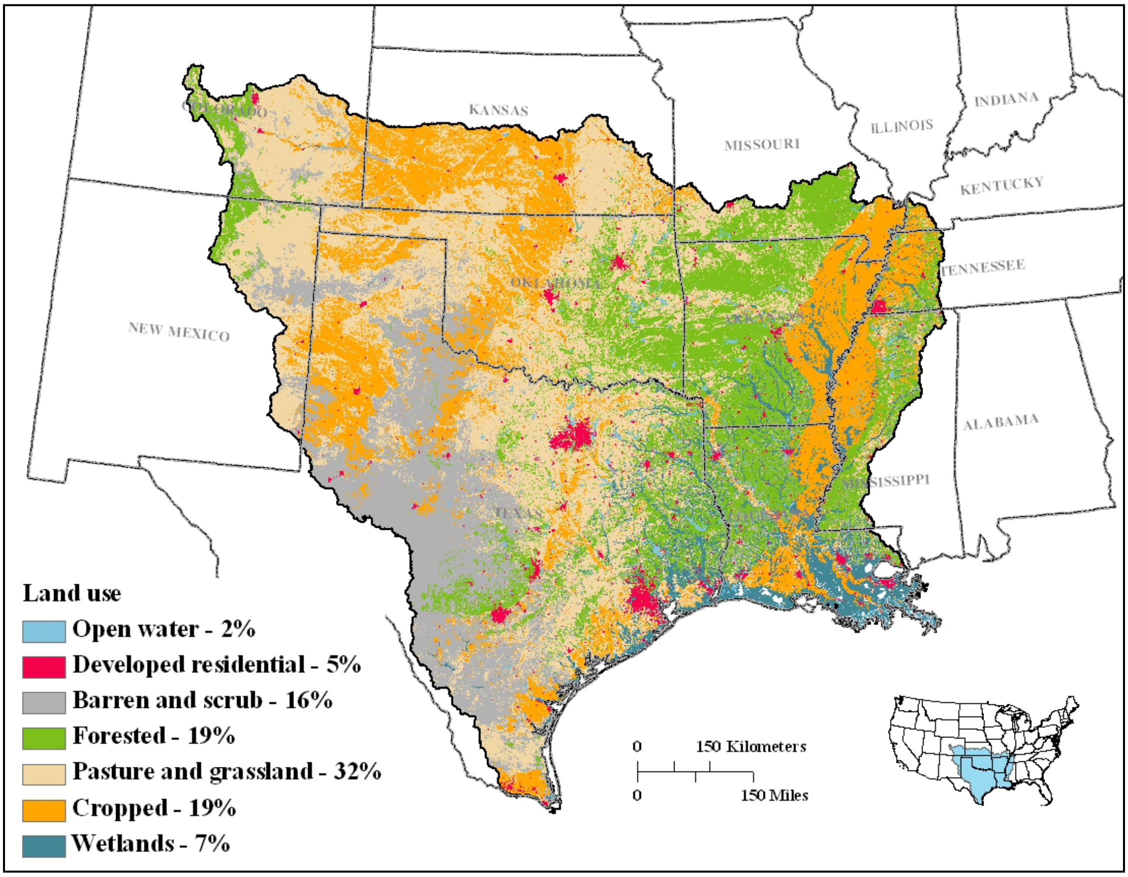

Figure S2 – Land use in the Lower Mississippi Texas-Gulf region.

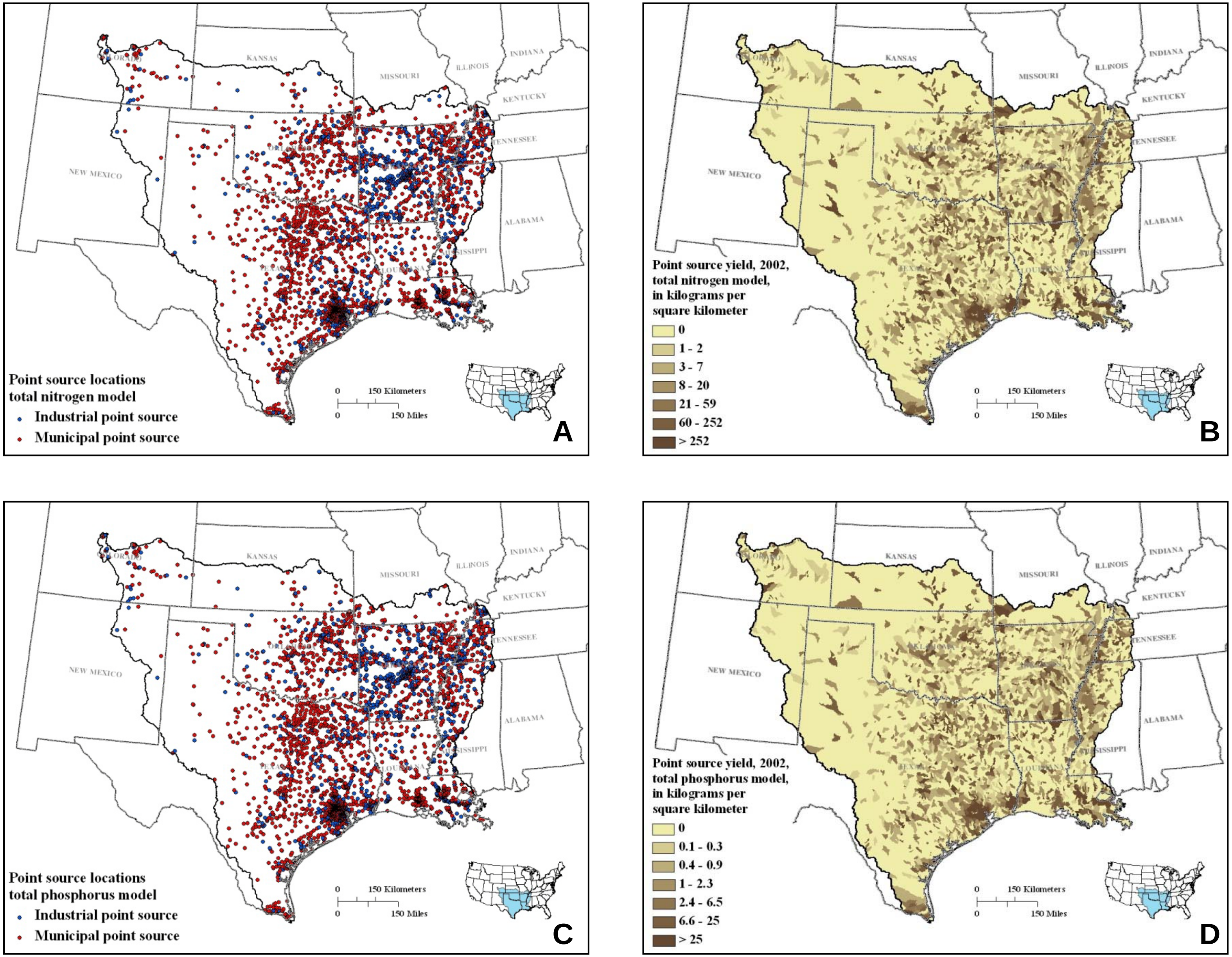

Figure S3 – 2002 point source information for the Lower Mississippi Texas-Gulf region: (a) total nitrogen point source locations and (b) yields; and total phosphorus point source (c) locations and (d) yields.

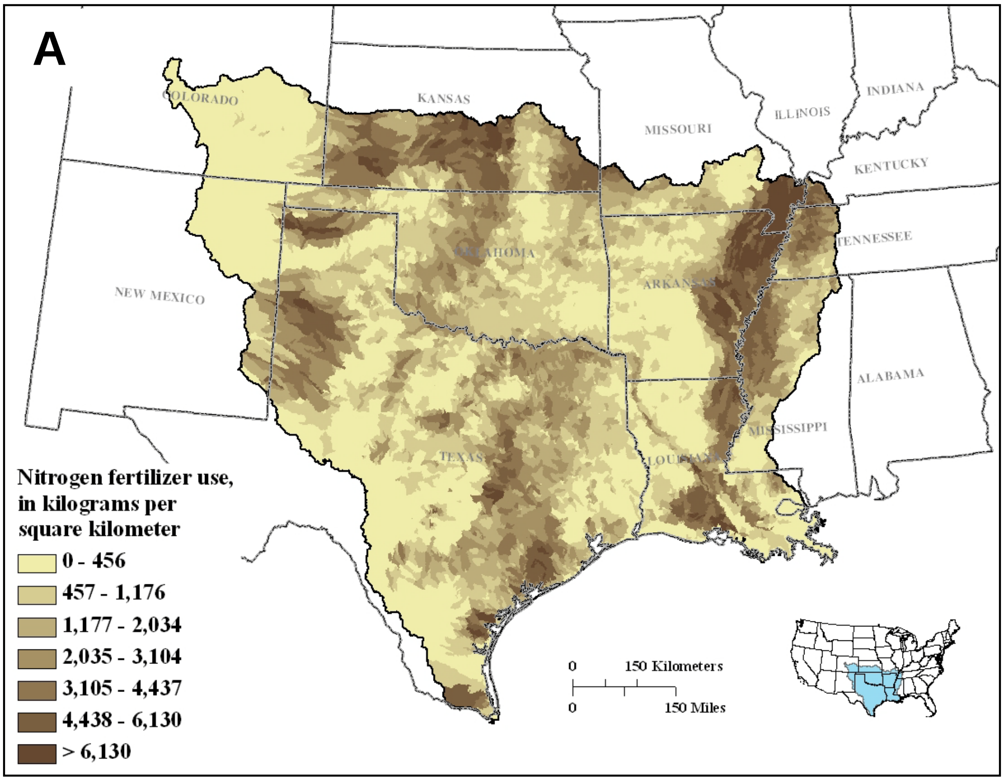

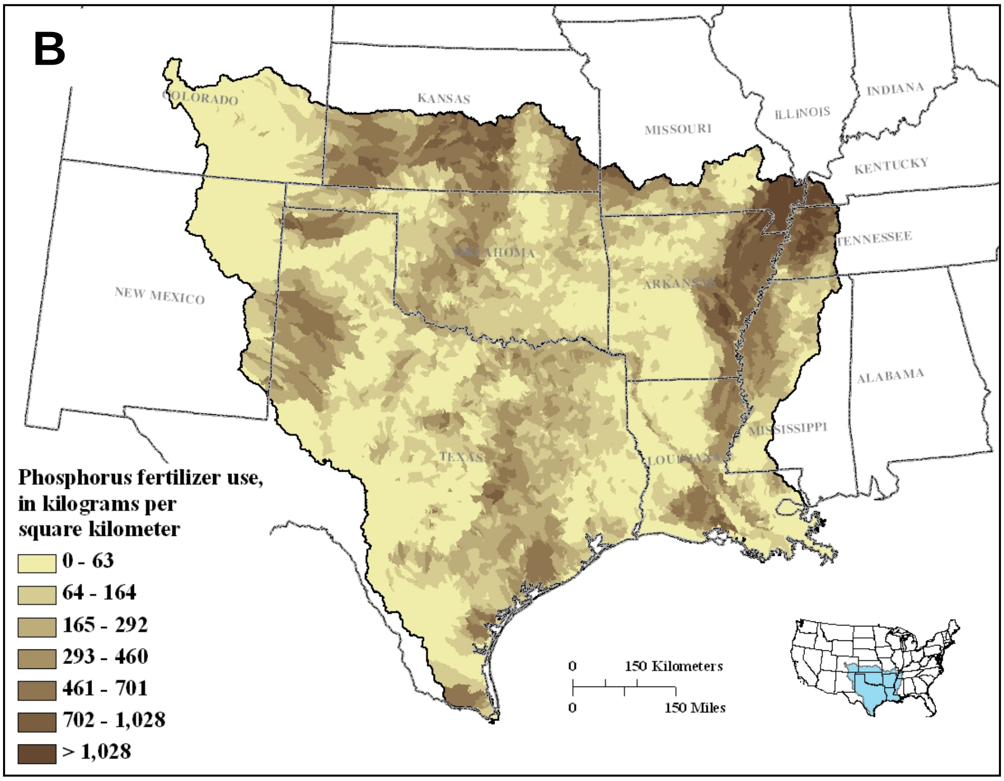

Figure S4 – (a) Nitrogen and (b) phosphorus fertilizer use by catchment in the Lower Mississippi Texas-Gulf region.

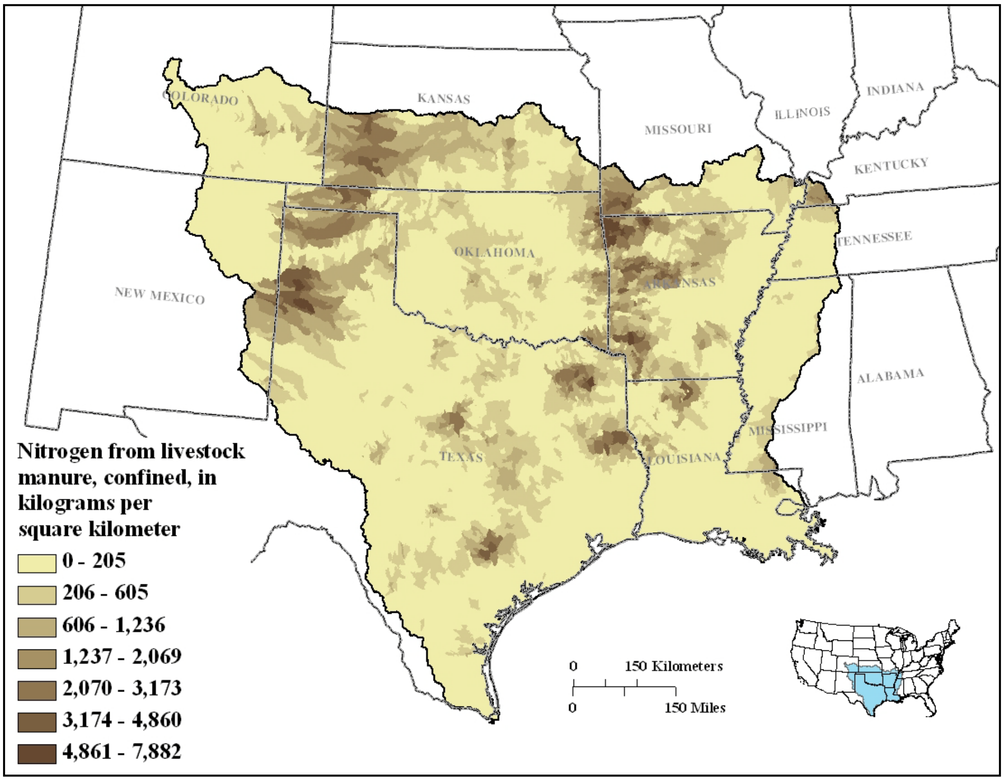

Figure S5 – Nitrogen from livestock manure from confined animal feeding operations for the Lower Mississippi Texas-Gulf region.

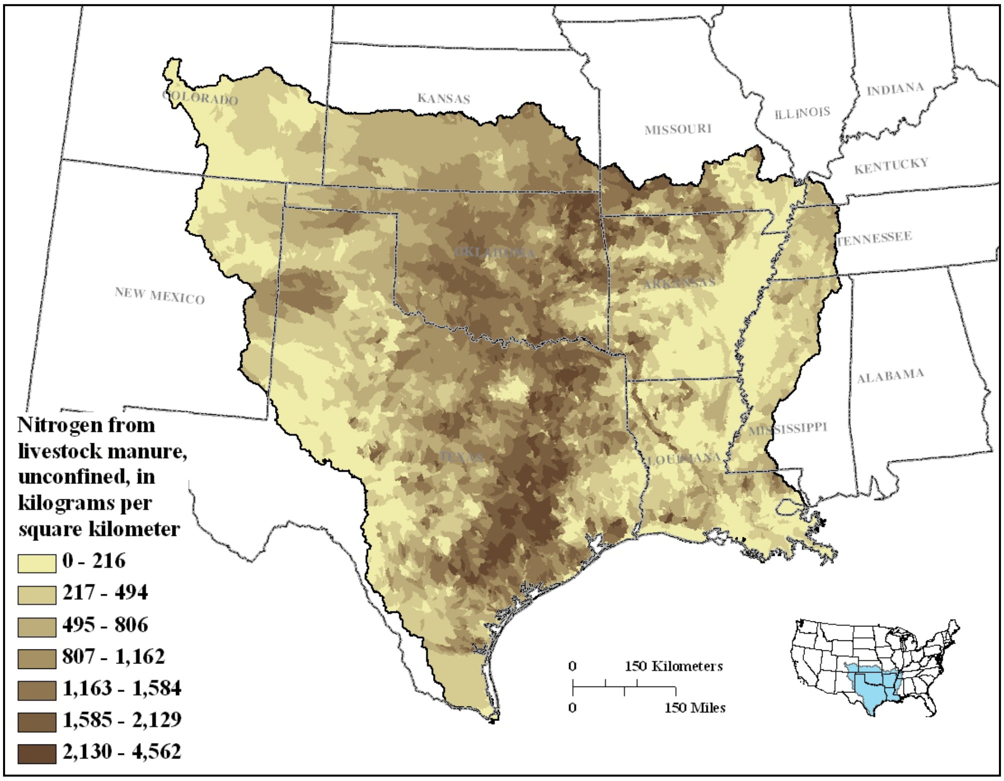

Figure S6 – Nitrogen from livestock manure from unconfined animal feeding operations for the Lower Mississippi Texas-Gulf region.

animal feeding operations used in the TN model. Figure S7 is a plot of the combined livestock manure data used in the TP model. Figure S8 is a plot of total inorganic N from wet deposition detrended to 2002 used as the atmospheric deposition dataset for the TN model. Figure S9 is a plot of channel length for reaches that have streamflow greater than 1.4 cubic meters per second in the LMTG region. Channel length was used as a surrogate for P attached to sediment caused by in-channel erosion in the TP model.

The 30-year average rainfall for the period 1971-2000, which was a highly significant land-to-water delivery term in both models is plotted in Figure S10. The western part of the region is fairly arid (annual rainfall less than about 70 cm total per year), and the eastern part has a humid, subtropical climate with annual rainfall amounts greater than about 100 cm per year. Figure S11 is a plot of overland flow in excess of infiltration, which was highly significant in both models. Overland flow in excess of infiltration was considered a surrogate for runoff potential in both models. Figure S12 is a plot of soil erodibility (or K-factor from the Universal Soil-Loss Equation) for the LMTG region, which was statistically significant as a land-to-water delivery term in the TP model.

MODEL RESULTS

Graphical evidence related to goodness-of-fit for each model is shown in Figure S13, where the natural log of the observed load is plotted with the natural log of the predicted load. These plots indicate that residuals for both models were normally distributed and homoscedastic (residuals are of constant variance and uniform scatter). The patterns in both plots for each model also indicate that the model has better accuracy

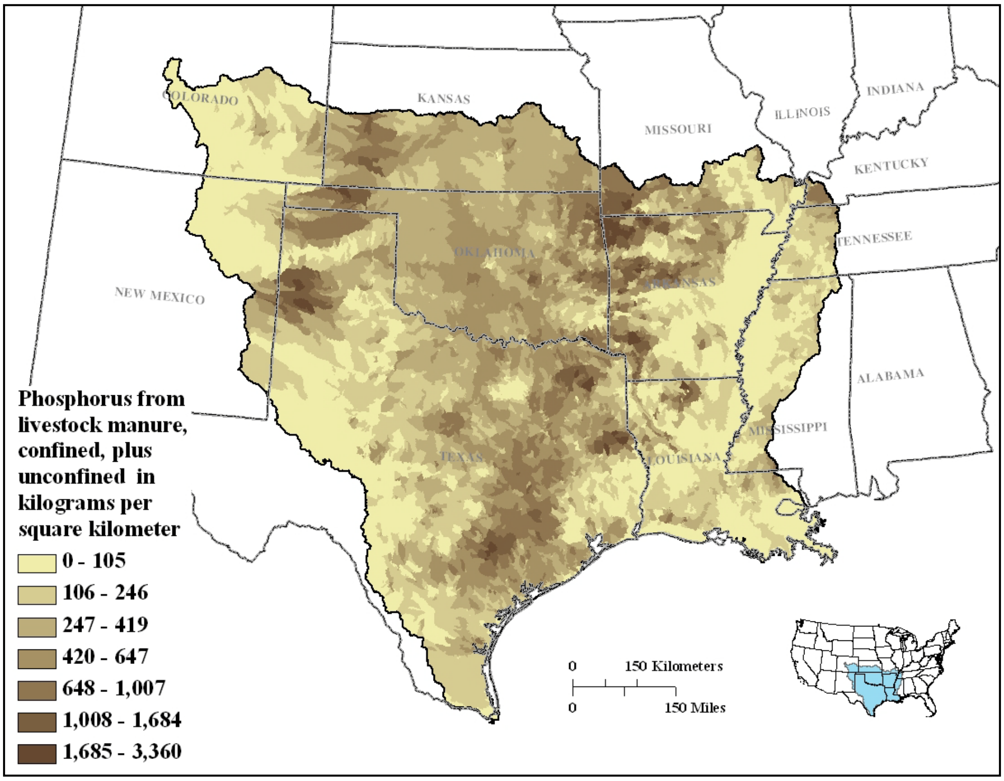

Figure S7 – Phosphorus from livestock manure from confined and unconfined animal feeding operations (combined source) for the Lower Mississippi Texas-Gulf region.

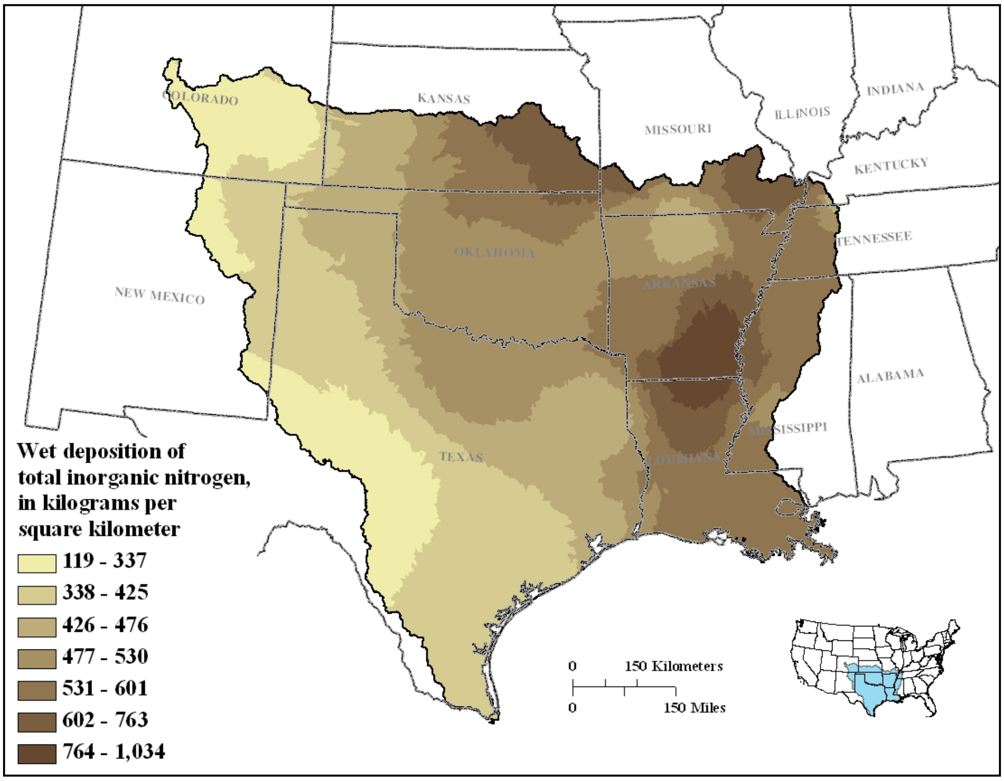

Figure S8 – Wet deposition of total inorganic nitrogen for the Lower Mississippi Texas-Gulf region.

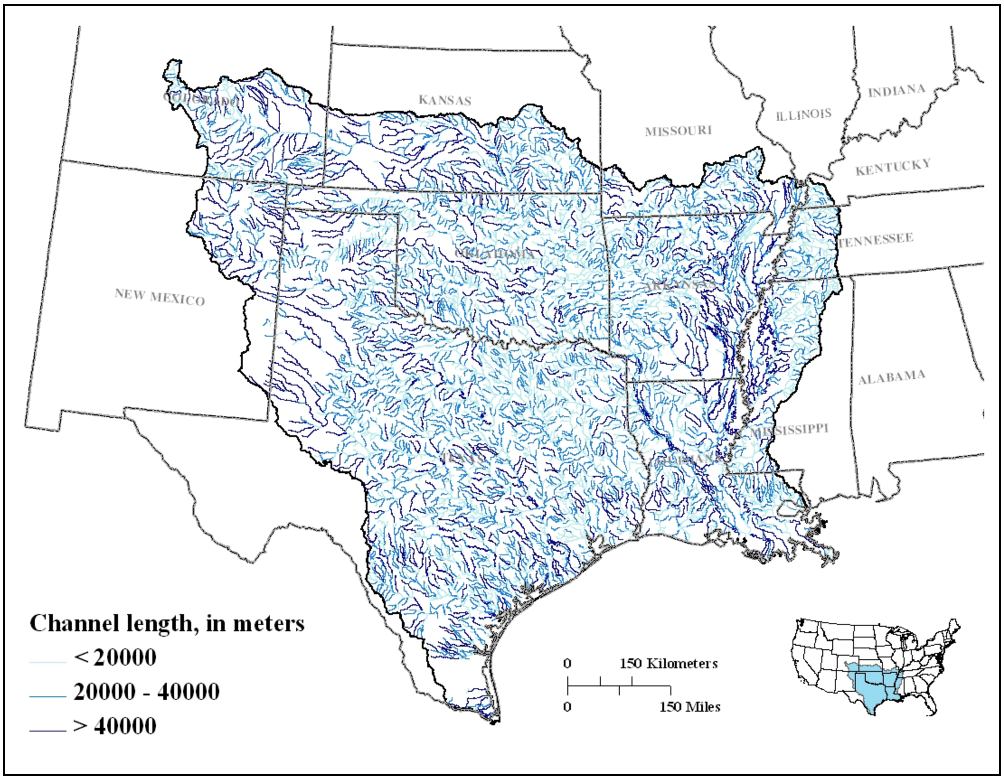

Figure S9 - Channel length for reaches that have streamflow greater than 1.4 m^3/s in the Lower Mississippi Texas-Gulf region. Channel length was used as a surrogate in the phosphorus SPARROW model to represent phosphorus attached to sediment caused by in-channel erosion.

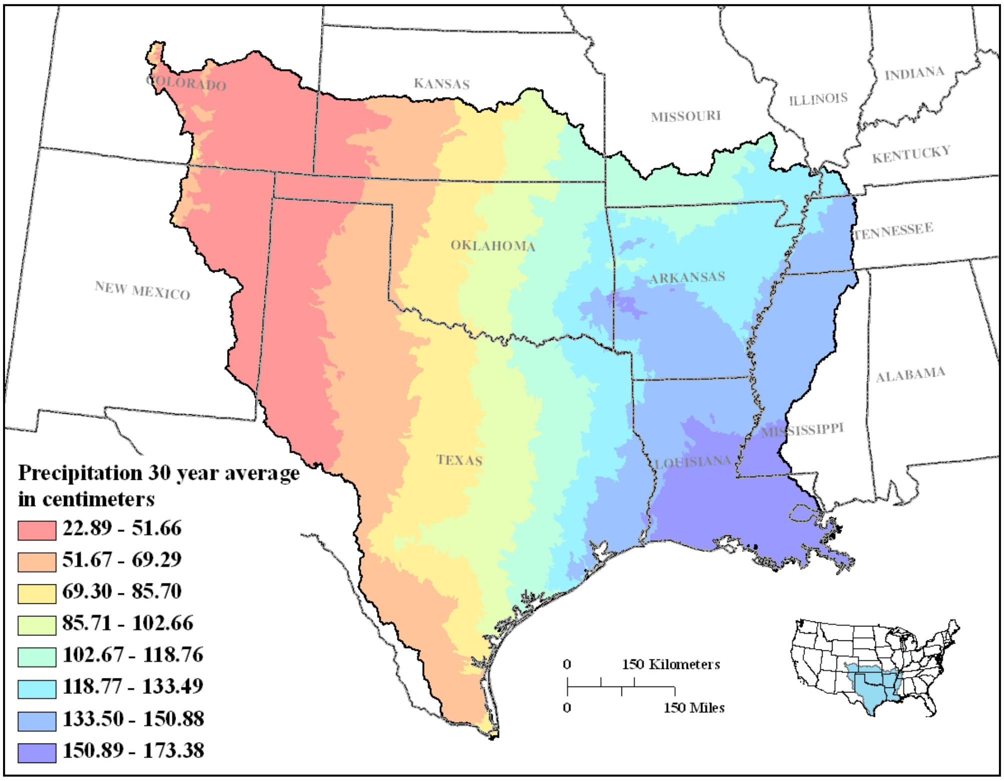

Figure S10 – 30-year average precipitation in the Lower Mississippi Texas-Gulf region.

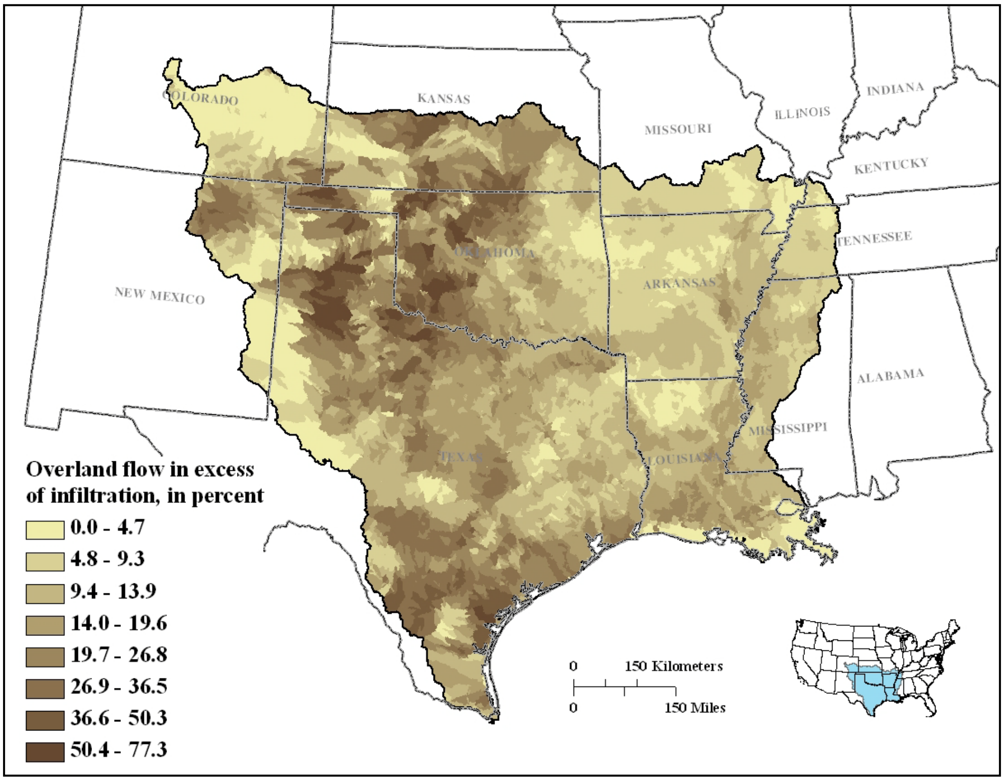

Figure S11 – Overland flow in excess of infiltration from TOPMODEL results. These data were statistically significant as a land-to-water delivery term in the total nitrogen and total phosphorus Lower Mississippi Texas-Gulf SPARROW models.

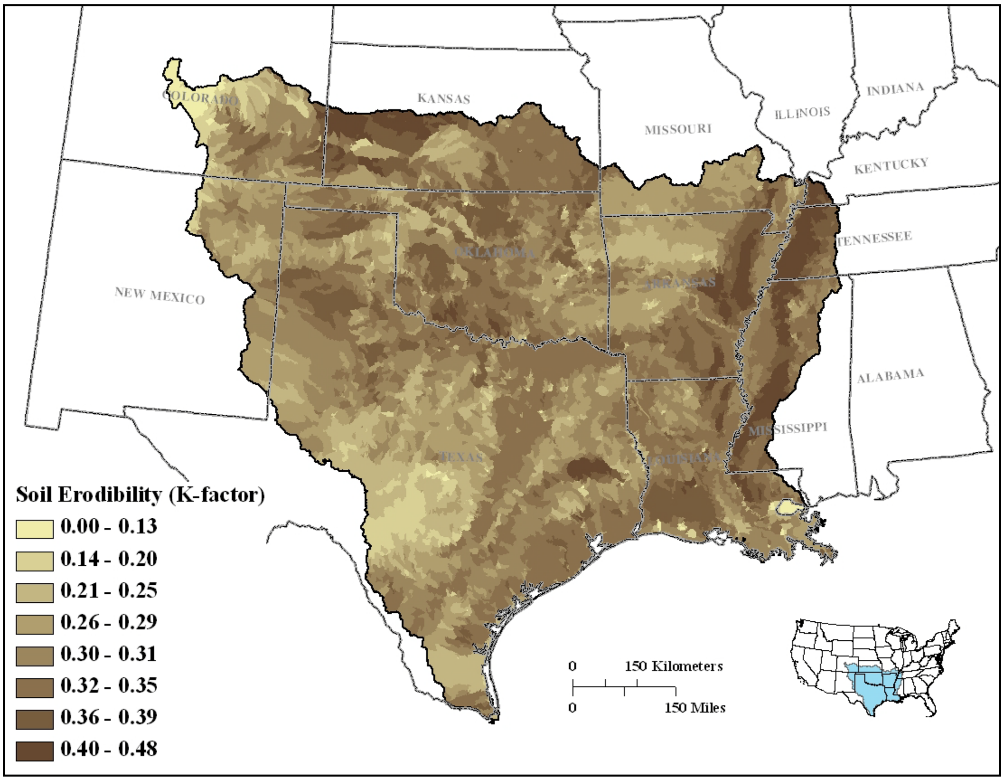

Figure S12 – Soil erodibility (K-factor from the Universal Soil-Loss Equation), which was statistically significant as a land-to-water delivery term in the Lower Mississippi Texas-Gulf total phosphorus SPARROW model.

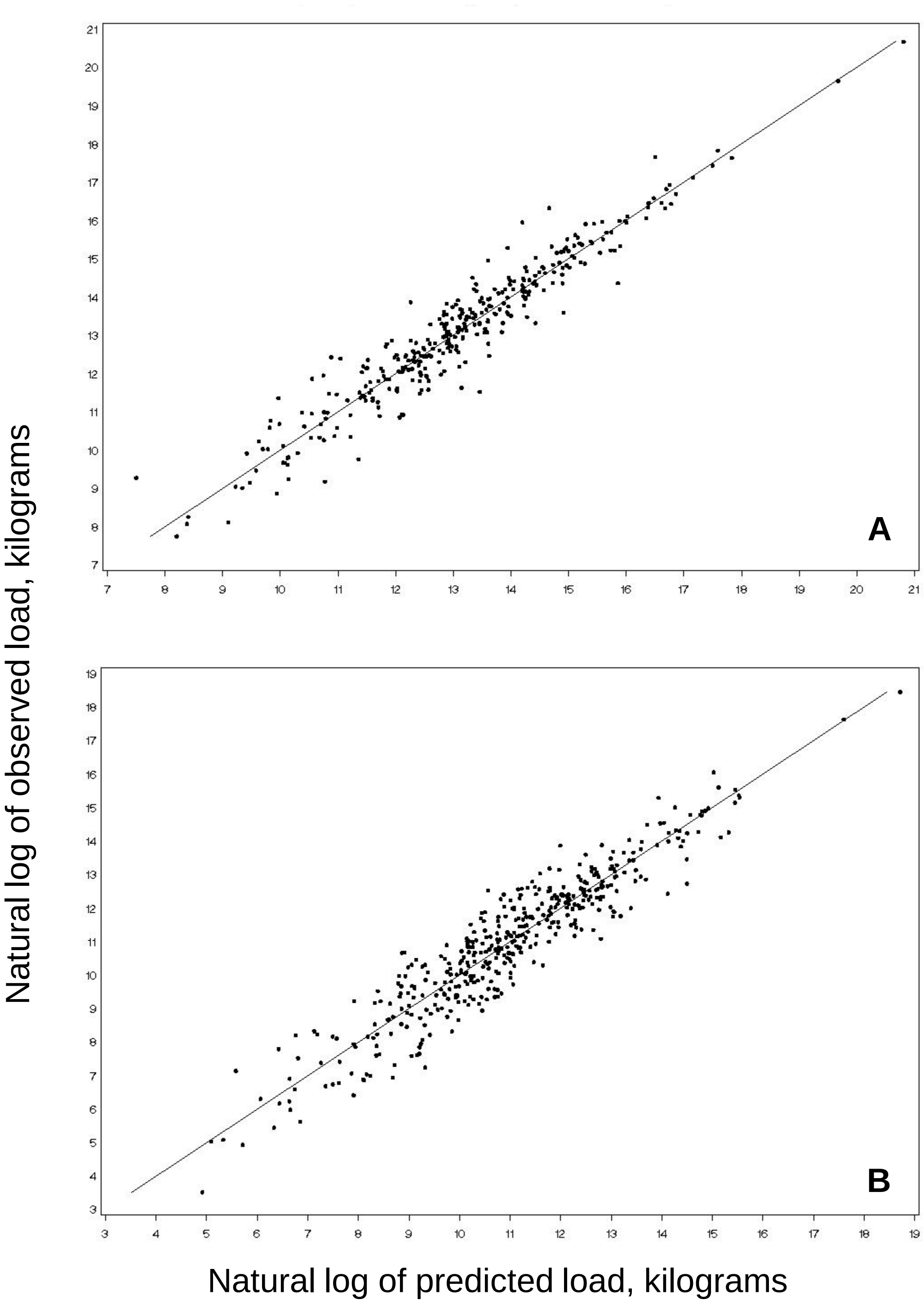

Figure S13. – Predicted loads plotted with observed loads for the Lower Mississippi Texas-Gulf (a) total nitrogen and (b) total phosphorus SPARROW models.

at sites with medium to large annual loads, and less accurate at sites with smaller loads. Figures S14a-n are maps of delivered incremental TN and TP yields for all LMTG watersheds, except for the Trinity River/Galveston Bay, which was presented in the article.

REFERENCES CITED IN SUPPLEMENTAL INFORMATION

Alexander, R.B., R.A. Smith, G.E. Schwarz, E.W. Boyer, J.V. Nolan, and J.W. Brakebill, 2008. Differences in phosphorus and nitrogen delivery to the Gulf of Mexico from the Mississippi River basin. *Environmental Science Technology*, 42:22-830.

Brakebill, J.W., D.M. Wolock, and S.E. Terziotti, this issue. Digital Hydrologic Networks Supporting Applications Related to Spatially Referenced Regression Modeling. Journal of the American Water Resources Association, doi: 10.1111/ j.1752-1688.2011.00578.x.

Judge, G.G., W.E. Griffiths, R.C. Hill, and T. Lee, 1985. The theory and practice of econometrics (2nd ed.): New York, NY, John Wiley and Sons, 1019 p.

Saad, D.A., G.E. Schwarz, D.M. Robertson, and N.L. Booth, this issue. A Multi-Agency Nutrient Dataset Used to Estimate Loads, Improve Monitoring Design, and Calibrate Regional Nutrient SPARROW Models. Journal of the American Water Resources Association, doi: 10.1111/j.1752-1688.2011.00575.x

SAS, 2003. SAS Statistical Software, SAS Institute Incorporated, Version 9.1.3 for Windows (Service Pack 3).

Schwarz, G.E., A.B. Hoos, R.B. Alexander, and R.A. Smith, 2006. The SPARROW surface water-quality model – theory, application, and user documentation: U.S. Geological Survey Techniques and Methods, Book 6, Section B, Chapter 3.

Smith, R.A., G.E. Schwarz, R.B.,Alexander, 1997. Regional interpretation of water-quality monitoring data. *Water Resources Research* 33(12):2781-2798.

U.S. Environmental Protection Agency (USEPA), 2004. STORET. http://www.epa.gov/STORET/index.html .

U.S. Geological Survey (USGS), variously dated. National field manual for the collection of water-quality data: U.S. Geological Survey Techniques of Water-Resources Investigations, book 9, chaps. A1-A9. http://pubs.water.usgs.gov/twri9A .

Wieczorek, M.E. and A.E. Lamotte. (2011). Attributes for MRB_E2RF1 Catchments by Major River Basins in the Conterminous United States. U.S. Geological Survey Digital Data Series DS-491. http://water.usgs.gov/nawqa/modeling/rf1attributes.html.

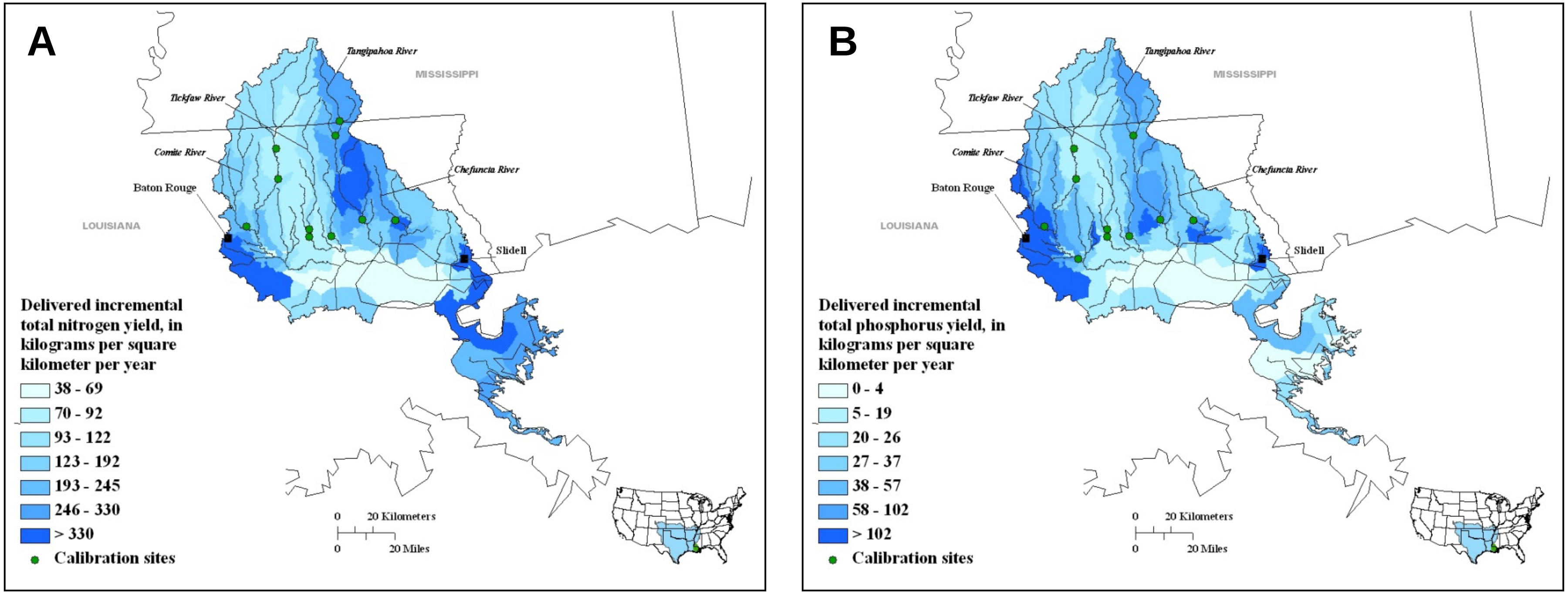

Figure S14A. – a) Delivered incremental total nitrogen yield and b) delivered incremental total phosphorus yield for the Lake Borgne watershed.

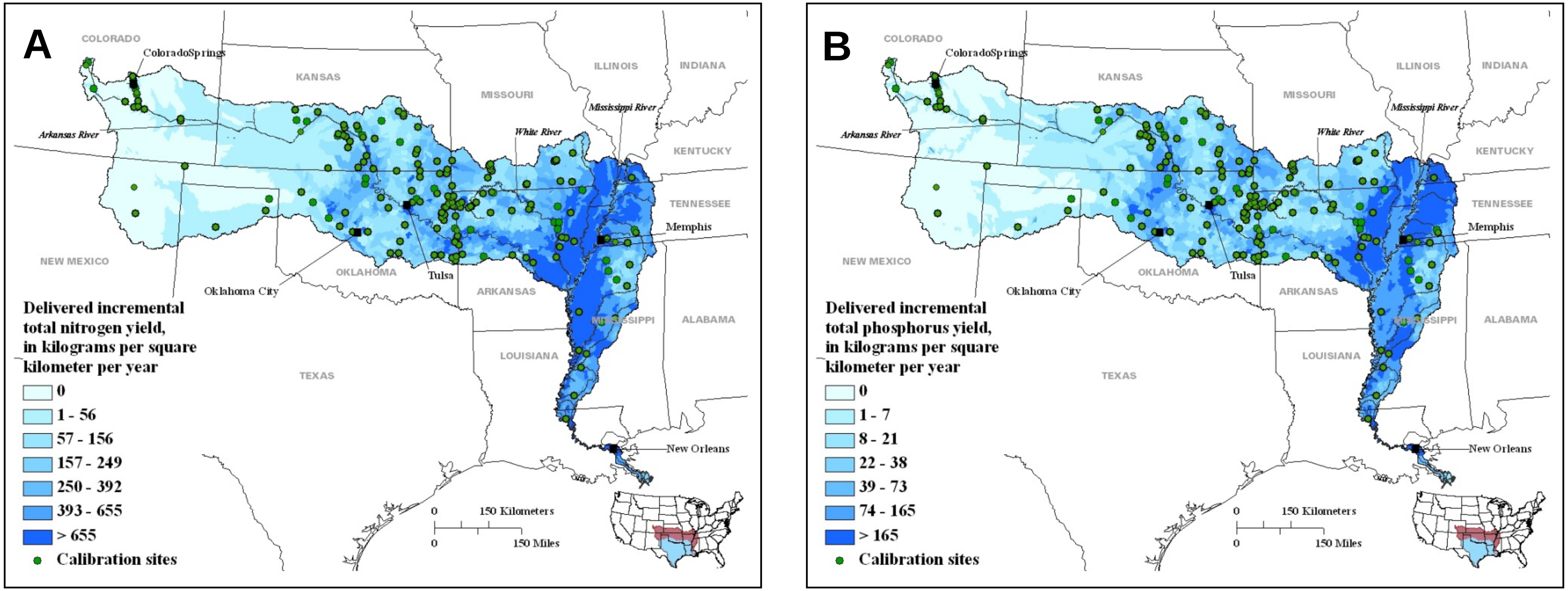

Figure S14B. – a) Delivered incremental total nitrogen yield and b) delivered incremental total phosphorus yield for the Mississippi River watershed.

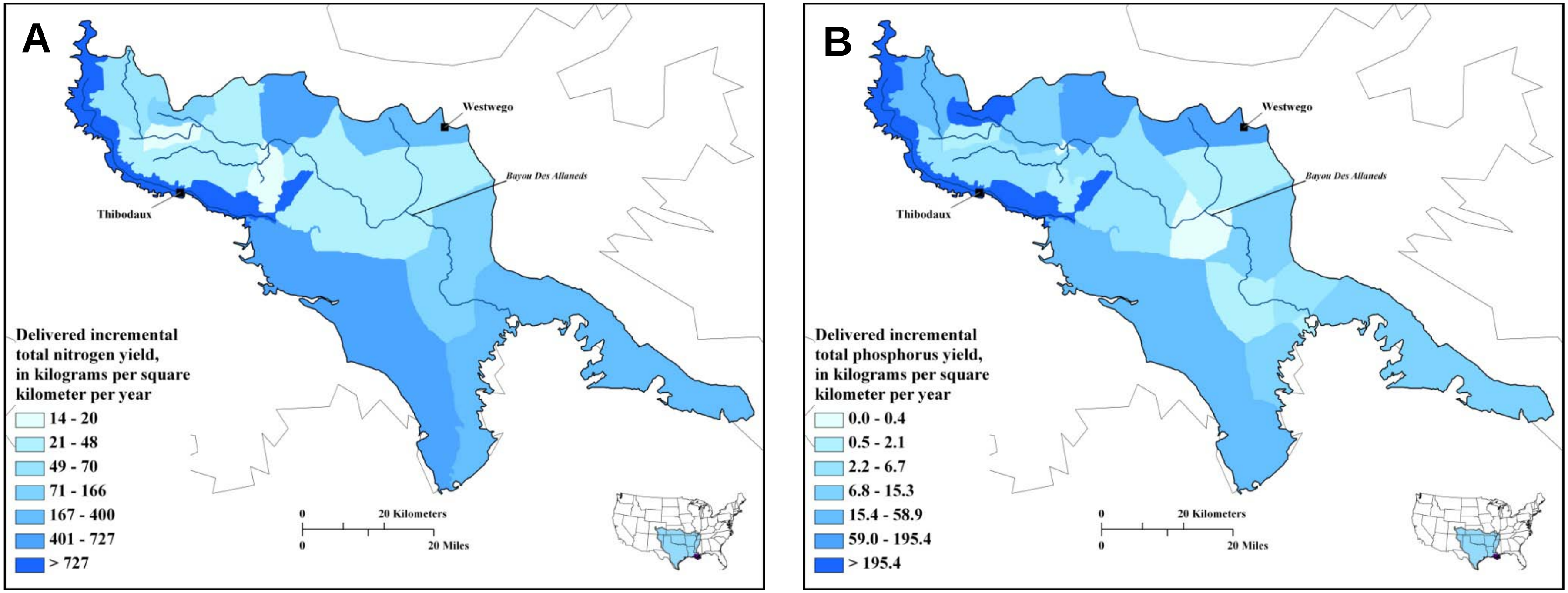

Figure S14C. – a) Delivered incremental total nitrogen yield and b) delivered incremental total phosphorus yield for the Barataria Bay watershed.

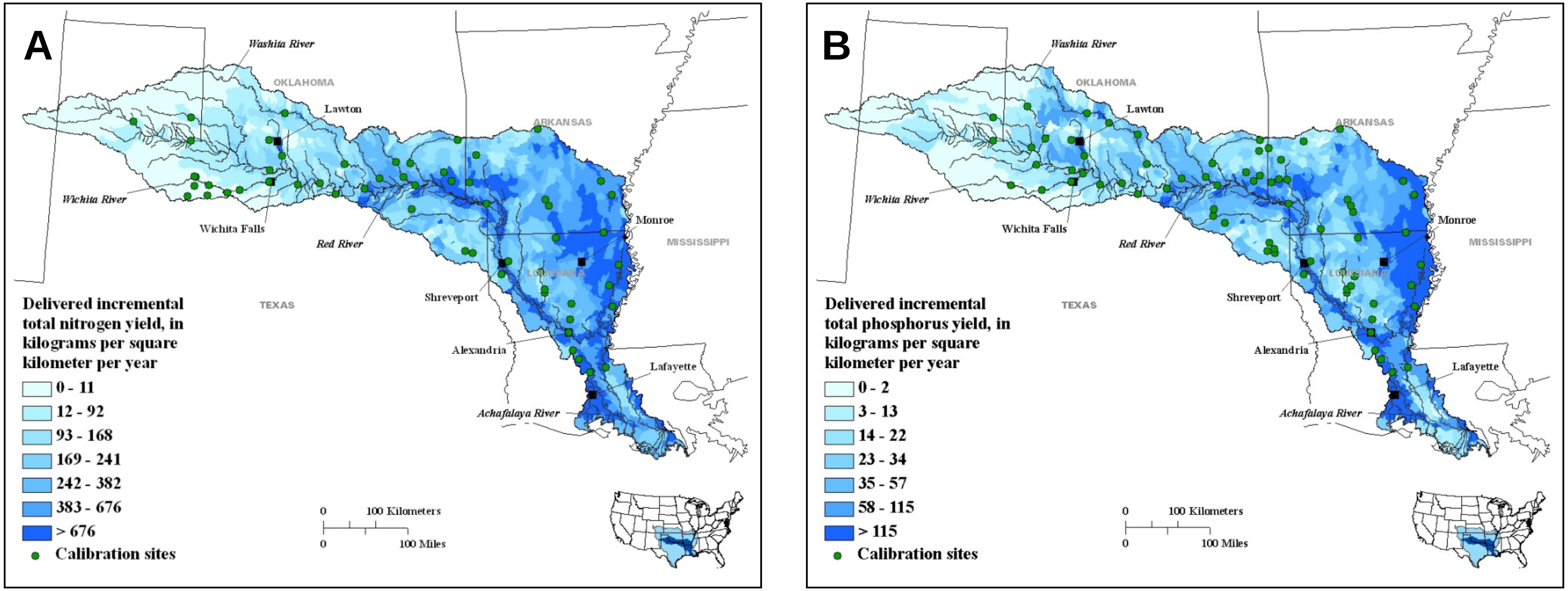

Figure S14D. – a) Delivered incremental total nitrogen yield and b) delivered incremental total phosphorus yield for the Atchafalaya River/Terrebonne Bay watershed.

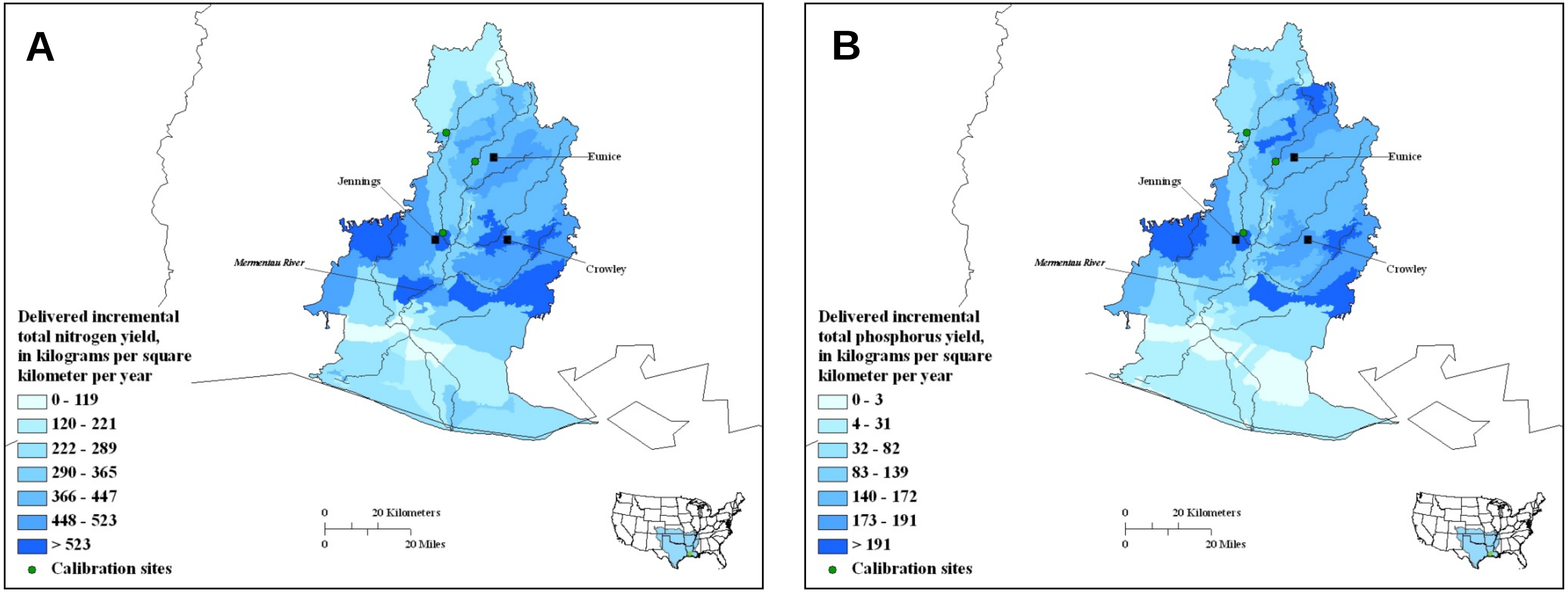

Figure S14E. – a) Delivered incremental total nitrogen yield and b) delivered incremental total phosphorus yield for the Mermentau River watershed.

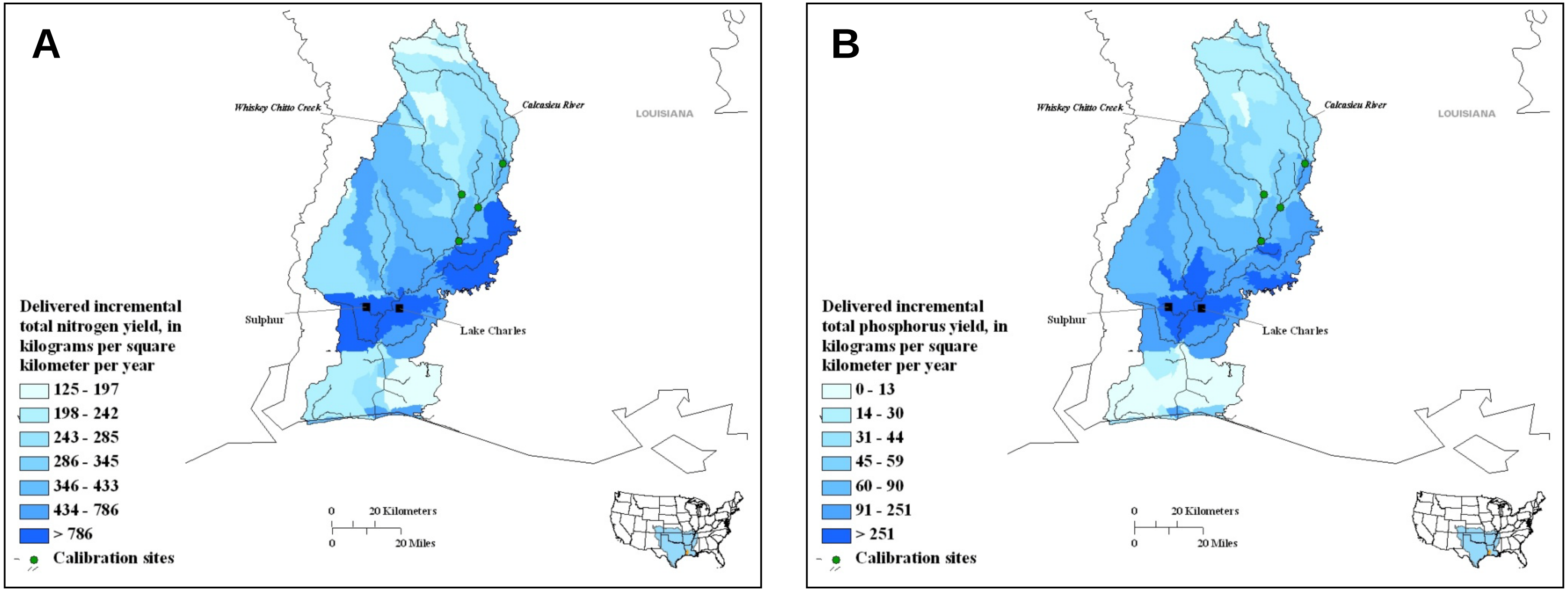

Figure S14F. – a) Delivered incremental total nitrogen yield and b) delivered incremental total phosphorus yield for the Calcasieu River watershed.

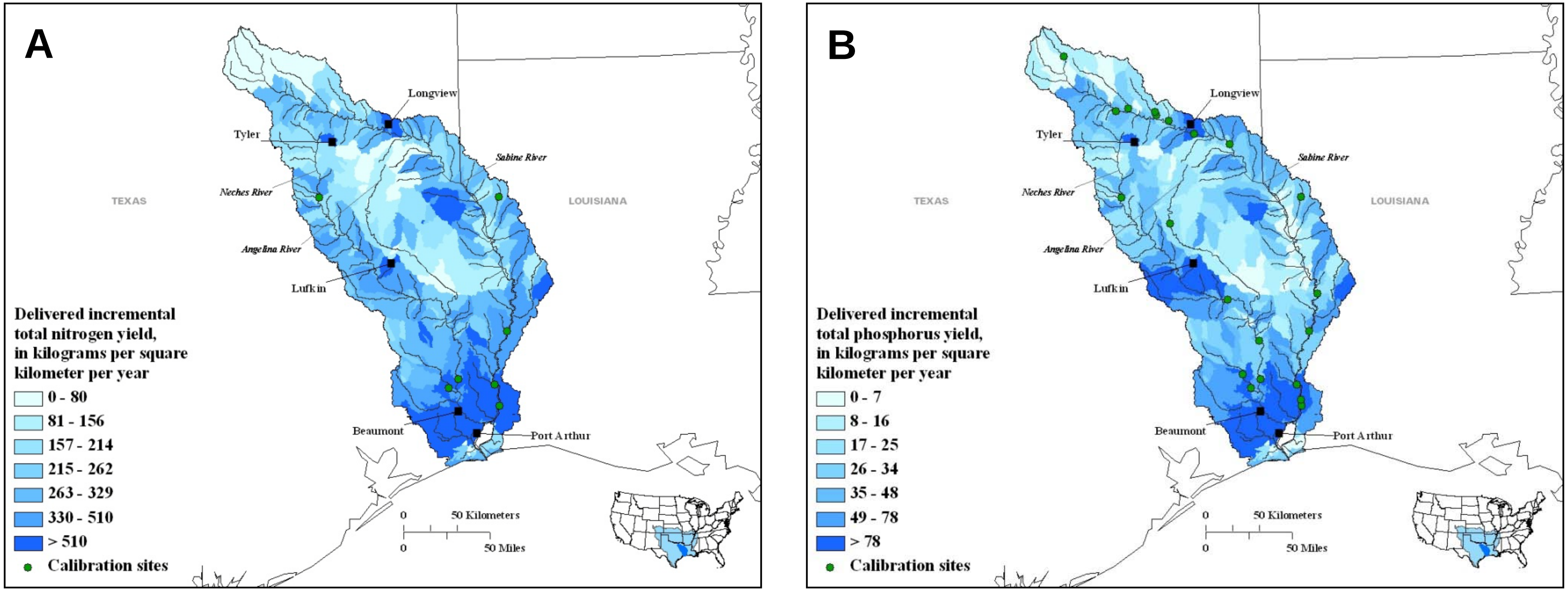

Figure S14G. – a) Delivered incremental total nitrogen yield and b) delivered incremental total phosphorus yield for the Neches/Sabine Rivers watershed.

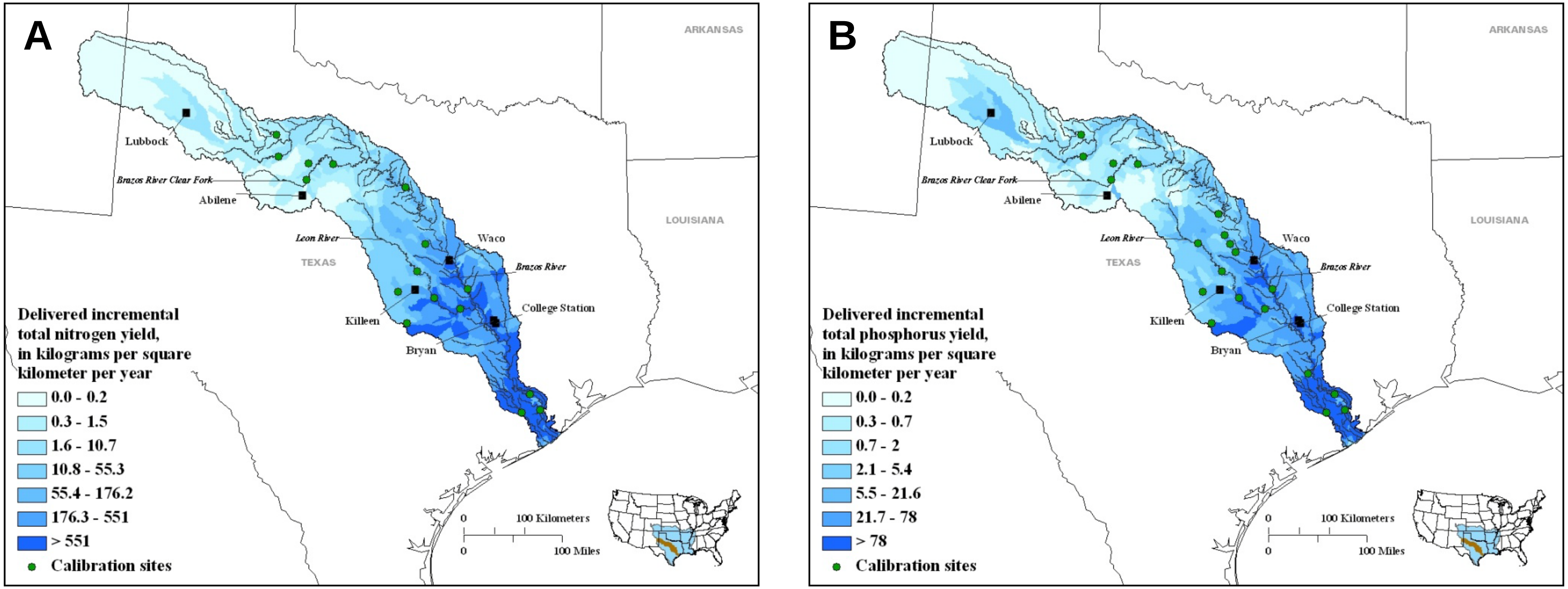

Figure S14H. – a) Delivered incremental total nitrogen yield and b) delivered incremental total phosphorus yield for the Brazos River watershed.

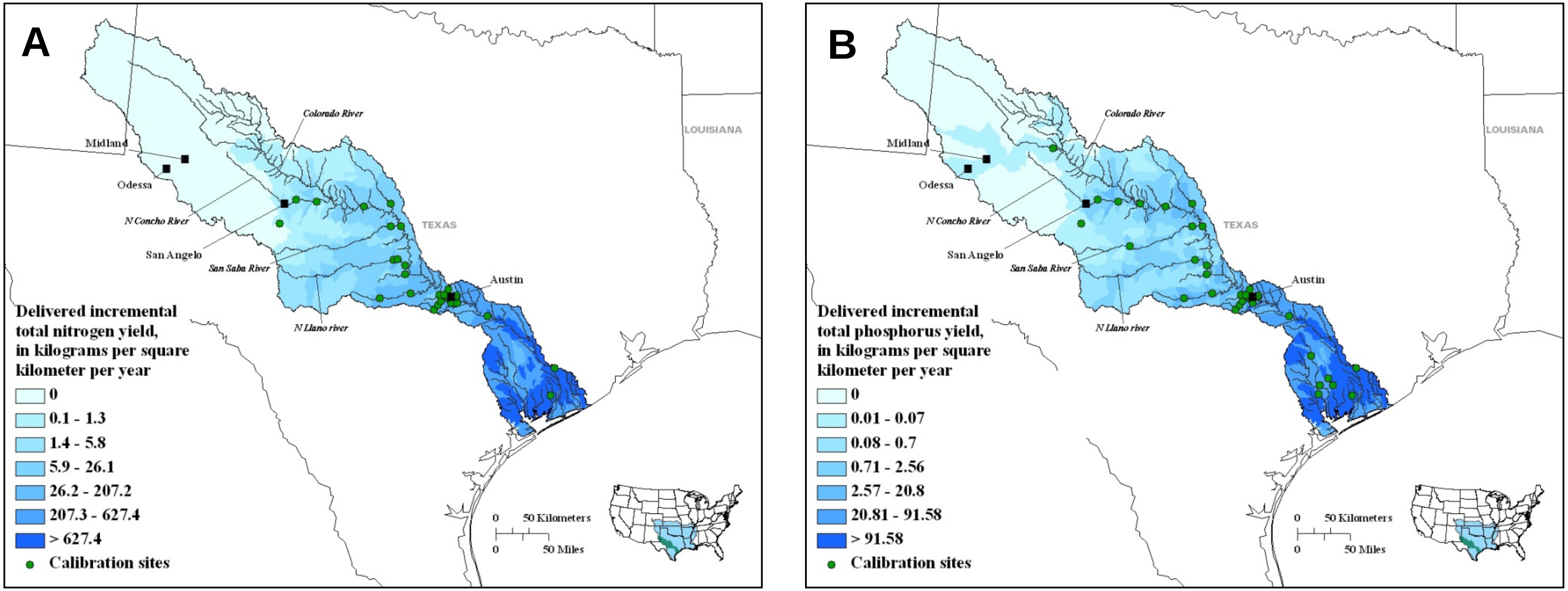

Figure S14I – a) Delivered incremental total nitrogen yield and b) delivered incremental total phosphorus yield for the Colorado River/Matagorda Bay watershed.

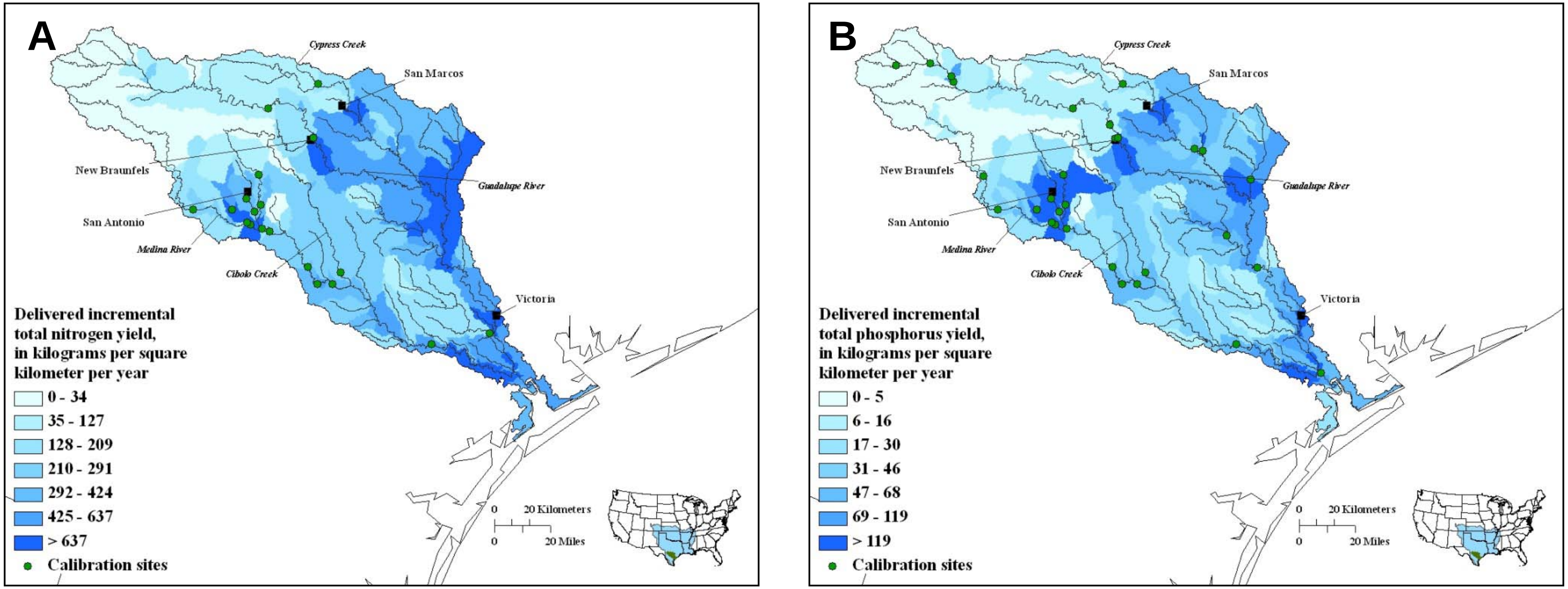

Figure S14J. – a) Delivered incremental total nitrogen yield and b) delivered incremental total phosphorus yield for the San Antonio/Guadalupe Rivers watershed.

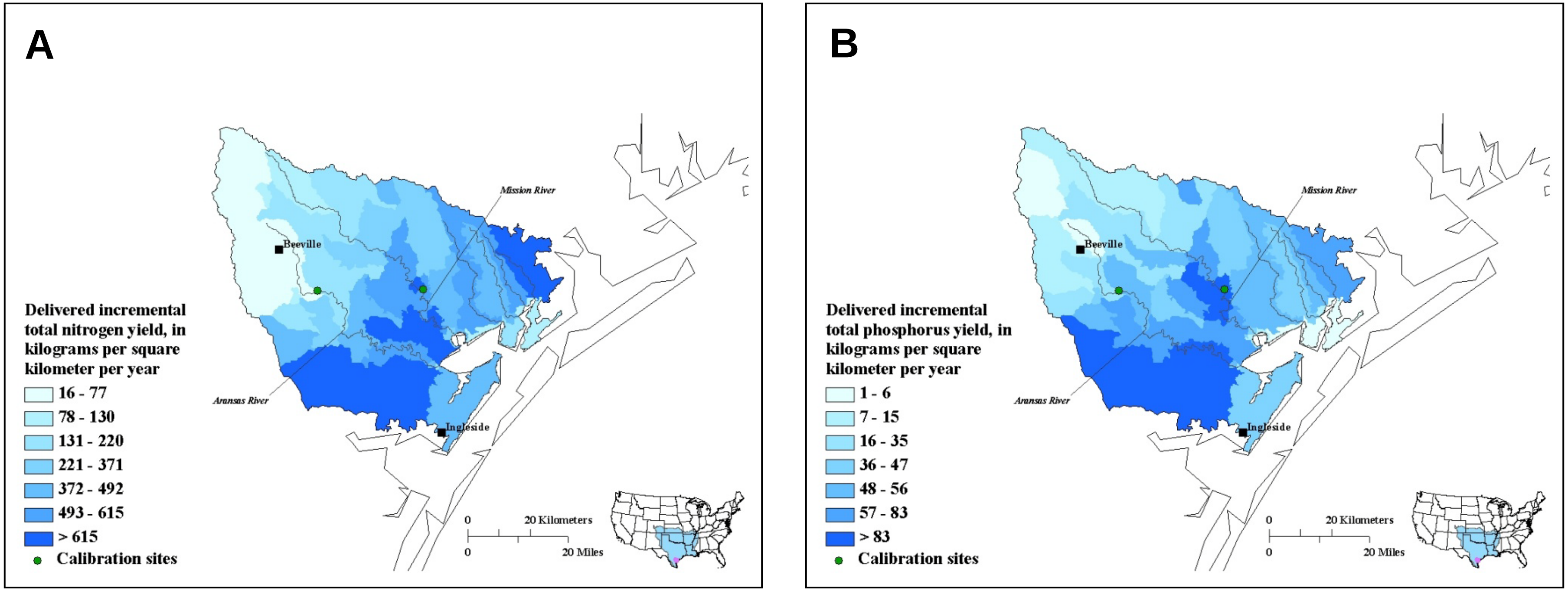

Figure S14K. – a) Delivered incremental total nitrogen yield and b) delivered incremental total phosphorus yield for the Aransas River watershed.

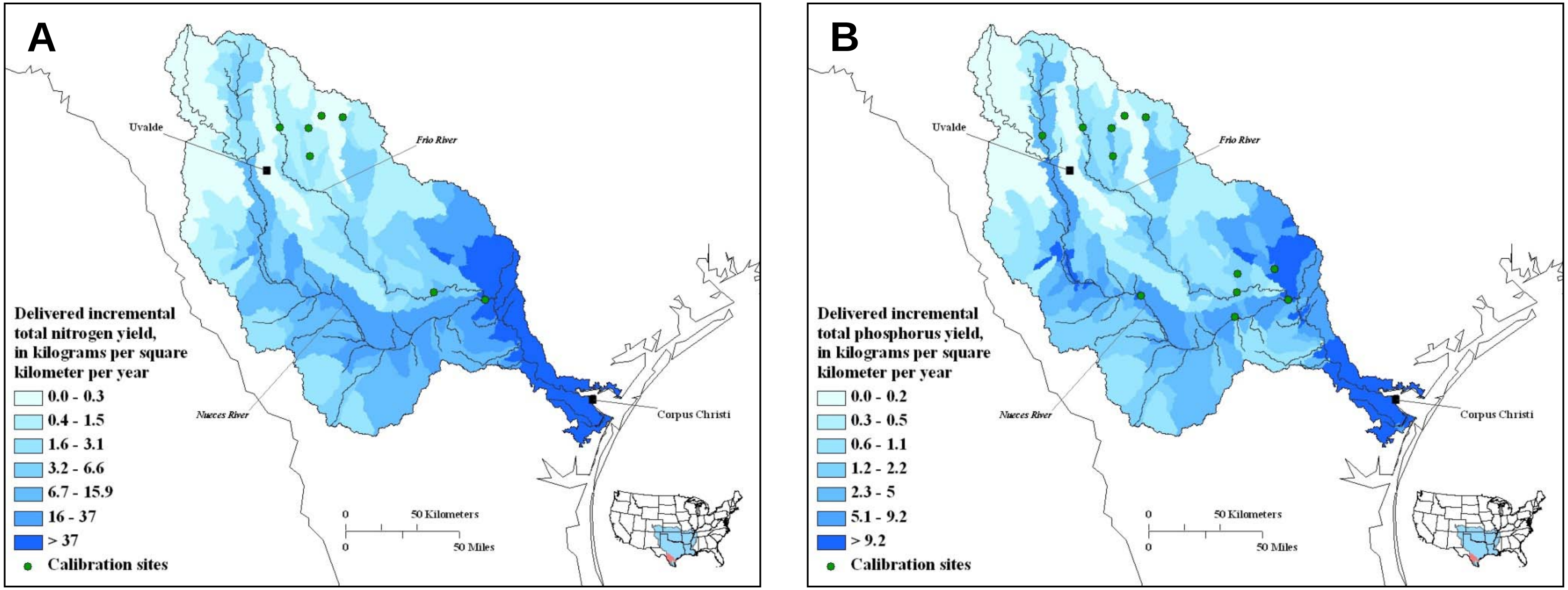

Figure S14L. – a) Delivered incremental total nitrogen yield and b) delivered incremental total phosphorus yield for the Nueces River/Corpus Christi Bay watershed.

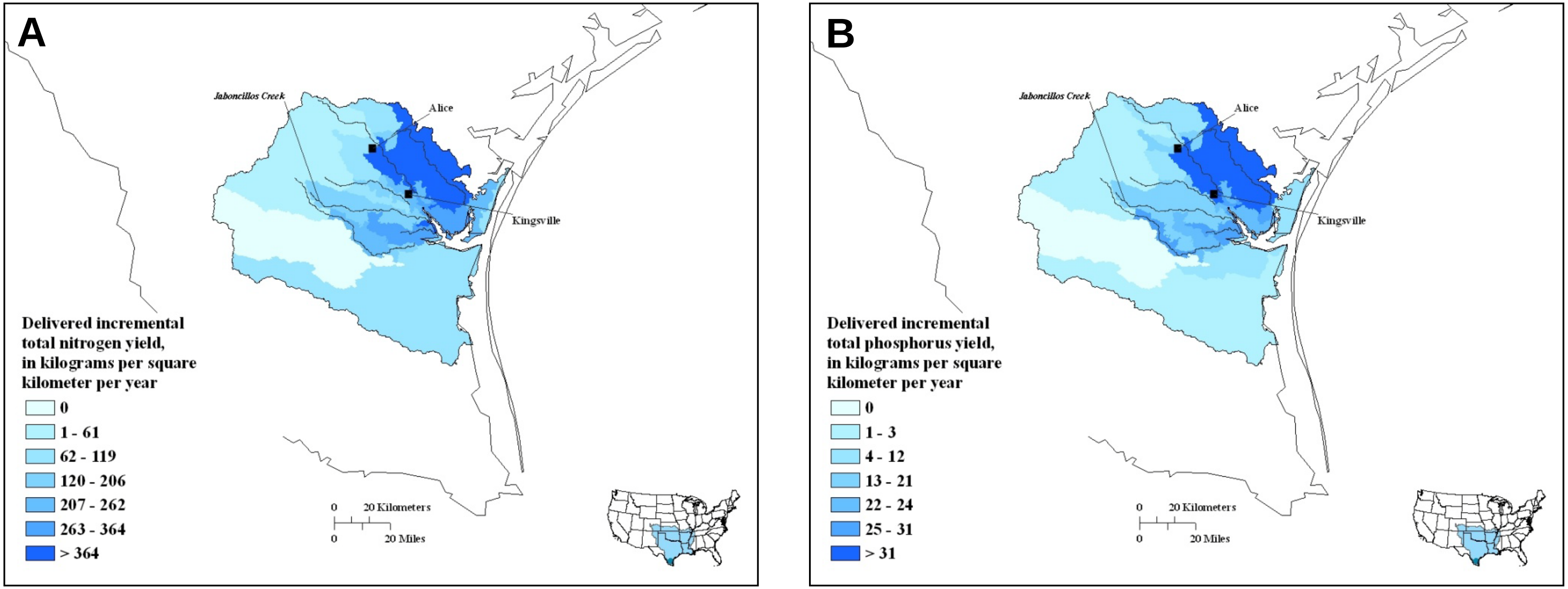

Figure S14M. – a) Delivered incremental total nitrogen yield and b) delivered incremental total phosphorus yield for the Upper Laguna Madre watershed.

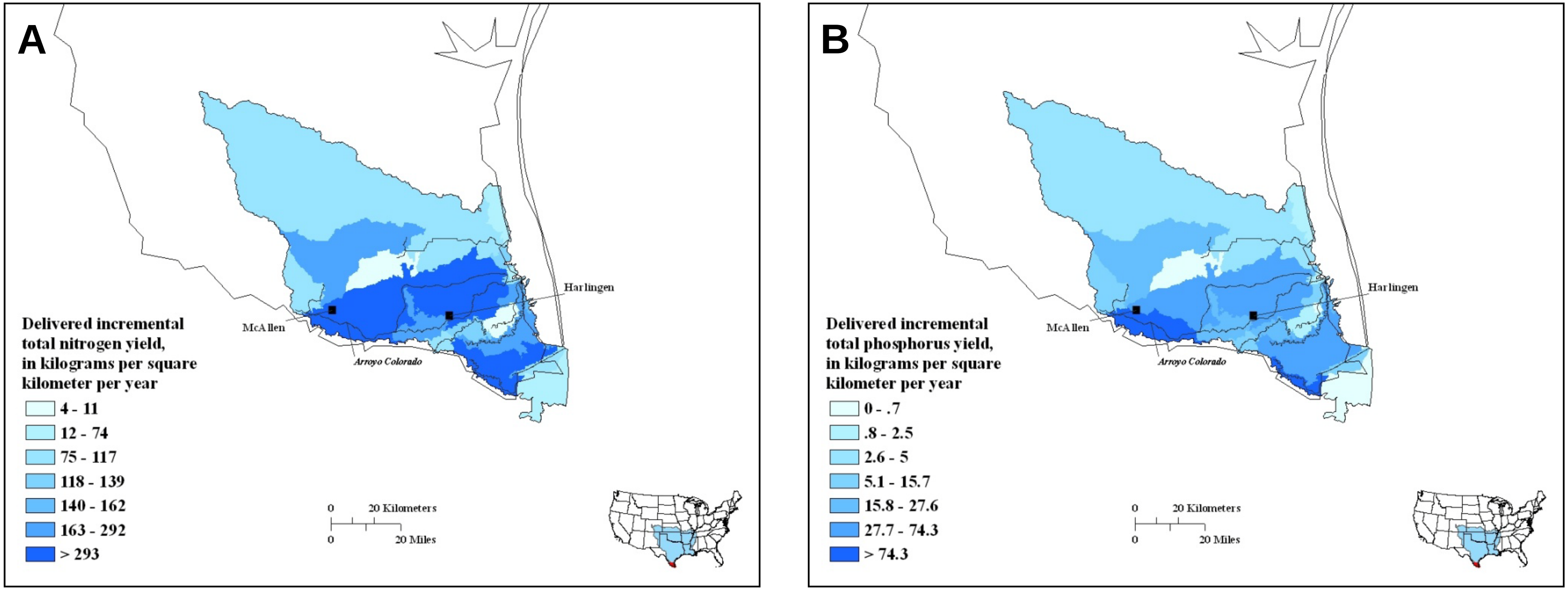

Figure S14N. – a) Delivered incremental total nitrogen yield and b) delivered incremental total phosphorus yield for the Lower Laguna Madre watershed.
